# Immunotherapy Bridge 2019 and Melanoma Bridge 2019: meeting abstracts

**DOI:** 10.1186/s12967-020-02209-y

**Published:** 2020-02-11

## Immunotherapy Bridge 2019

### SITC session—mechanisms of success and failure in immunotherapy

#### Oral communications

##### 1 Gal9/Tim-3 expression level is higher in patient with failed chemotherapy in AML

###### Paola Dama, Marshall Tang, Noreen Fulton, Justin Kline, Hongtao Liu

####### University of Chicago Medicine Hematology/Oncology Section, 5841 S Maryland Ave, Chicago, IL, 60637, USA

**Correspondence:** Paola Dama - dmapla@gmail.com, p.dama@sussex.ac.uk; Hongtao Liu - hliu2@medicine.bsd.uchicago.edu

*Journal of Translational Medicine* 2020, **18(Supp 1)**:1

**Background:** Activation of immune checkpoint pathways in Acute Myeloid Leukemia (AML) may interfere with effective T-cell anti-tumor immunity, and is associated with immune evasion in pre-clinical leukemia models as it has been demonstrated [1, 2]. It was previously reported that overexpression of CTLA4 and PD-1 is associated with more aggressive leukemia and progression from MDS to AML or AML relapse. While PD-1/PD-L1 blockade therapy can be effective as cancer immunotherapy, interruption of PD-1/PD-L1 interactions alone does not completely restore T cell function in some patients indicating the involvement of additional negative regulatory pathways, such as Tim-3/Gal-9, in T cell exhaustion. Immune checkpoint pathways active in Acute Myeloid Leukemia (AML) patients, especially during the course of remission induction chemotherapy, have not been well-studied. We characterized these pathways in newly diagnosed AML patients enrolled in a phase I dose escalation trial that combined Selinexor a Selective Inhibitor of Nuclear Export (SINE) with high-dose cytarabine (HiDAC) and mitoxantrone (Mito) (NCT02573363) as induction therapy.

**Methods and study design:** Multi-parameter flow-cytometry was performed on bone marrow specimens at diagnosis and following remission induction therapy in 26 patients with AML enrolled to the study to monitor the changes in expression of immune checkpoint receptors. Expression of CD47, PD-L1, PD-L2 and Gal-9 was assessed on CD34+ AML blasts and CD34- cell populations. In parallel, expression of inhibitory (PD1, CTLA4, LAG3, TIM3) and stimulatory co-receptors (CD28, ICOS, CD137, OX40, CD40L, HLA-DR) on CD4+ and CD8+ T cell subsets were evaluated. The positivity and frequency of parent in percentage of each markers was gauged by comparing with their FMO controls. Samples were analyzed using LSR Fortessa or LSRII Cytometers. The Mann–Whitney Test, Spearman’s rank correlation and Runs Test analysis were applied. For all analyses, P-values < 0.05 were considered statistically significant.

**Results:** The percentage of CD34− Gal9+ cells was significantly higher and was positively correlated with higher numbers of TIM-3-expressing T cells at the time of diagnosis in patients who experienced treatment failure (TF) after chemotherapy, compared to those in complete remission (CR). When comparing TIM-3 expression on CD4+ and CD8+ T cells in pre-treatment (diagnosis) to post induction therapy samples, the magnitude of increase measured by median fluorescence intensity (MFI) inversely correlated to response to therapy with increase TIM-3 MFI of > 50% in patients with TF.

**Conclusions:** This study provides preliminary evidence to support a rationale for incorporating antibodies against the Gal9/TIM3 pathway during and/or following remission induction therapy for AML.

**References**

1. Zhang L, Gajewski TF, Kline J, PD-1/PD-L1 interactions inhibit antitumor immune responses in a murine acute myeloid leukemia model. Blood. 2009; 114(8):1545–52.
2. Zhou Q, Munger ME, Blazar BR, Coexpression of Tim-3 and PD-1 identifies a CD8+ T-cell exhaustion phenotype in mice with disseminated acute myelogenous leukemia. Blood. 2011;117(17):4501–10.

The study was approved by the Institutional Review Board at The University of Chicago (IRB15-0412) (Fig. 1).

**Fig. 1:**
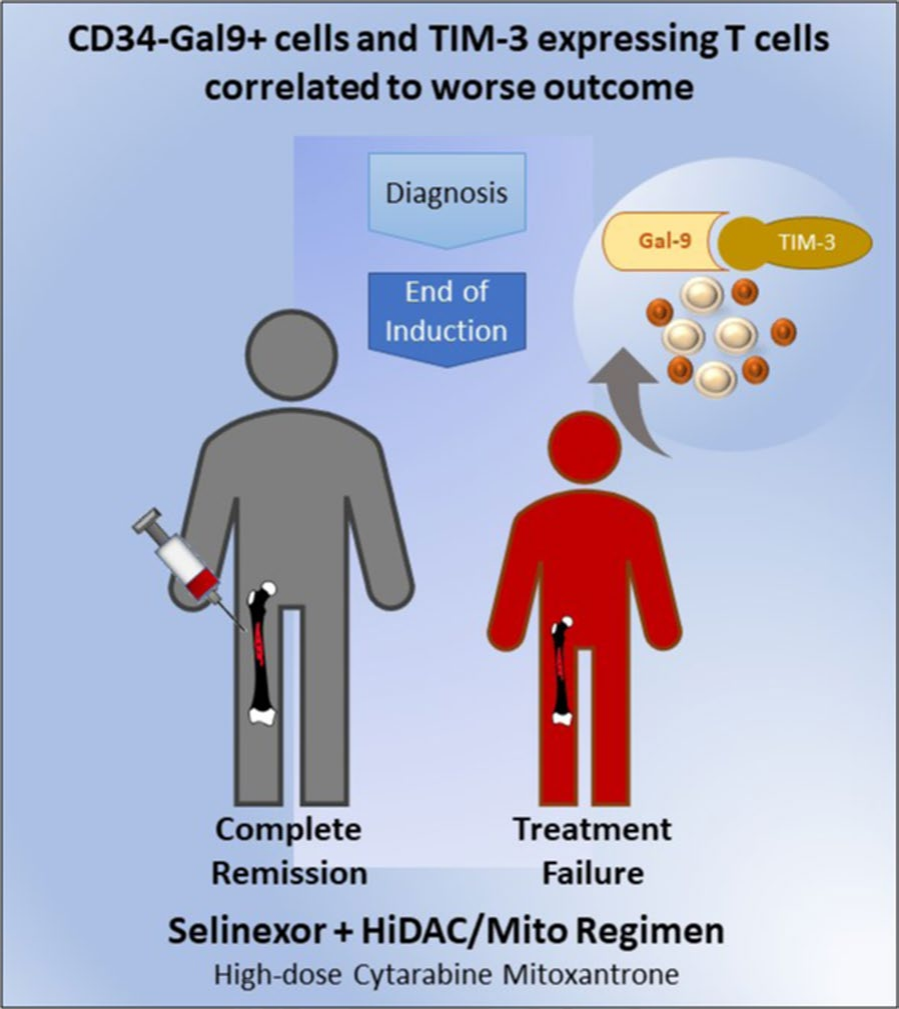
Visual abstract

##### 2 Gender differences in prognostic value of immune-related biomarkers in colon cancer patients randomized to surgery or surgery and adjuvant chemotherapy treatment

###### Lisa Villabona, Jacob Karlsson, Giuseppe Masucci, Peter Ragnhammar

####### Dept of oncology/pathology, Karolinska Institutet, Stockholm, Sweden

**Correspondence:** Lisa Villabona - Lisa.villabona@ki.se

*Journal of Translational Medicine* 2020, **18(Supp 1)**:2

**Background:** HLA-A*02, a common allele in the Scandinavian population, is a negative prognostic factor in epithelial ovarian cancer. It is a strong predictor of patient outcome, only inferior to clinical staging. This prognostic trait in epithelial ovarian cancer is stronger by the presence of the gene compared with the expression of its protein, MHC class I. Microsatellite instability (MSI) is used as a biomarker for prognosis and is suggested an increased tumor mutational burden which can make the tumor more susceptible for T cell mediated immunotherapy. Our aim was to analyze the prognostic markers HLA-A*02 genotype, MHC class I on tumor cells, the CD8+ lymphocyte infiltration and MSI status in colon cancer patients with randomized treatment.

**Methods:** Clinical information and primary tumors were collected from 520 colon cancer patients and followed for overall survival for 120 months. Patients hade stage II and III colon cancer and were randomized to surgery alone or surgery and adjuvant chemotherapy. HLA-A*02 genotype was determined by conventional PCR. MHC class I, MSI status and CD8+ lymphocyte infiltration were determined by immunohistochemistry.

**Results:** Female patients with a stage III tumor and HLA-A*02 genotype had a better outcome if they had received adjuvant chemotherapy instead of just surgery (p = 0.03), whereas this was not the case for patients with other HLA-A genotypes or in the male patients where HLA-type did not correlate to outcome. MHC class I expression did not act as a prognostic factor, however the presence of CD8+ lymphocytes in the invasive margin and inside the tumor was a positive prognostic factor for overall survival (p = 0.01), although only statistically significant in the male patients (p = 0.03). 21% patients had a tumor with MSI (23% of the female and 19% of the male patients respectively). MSI tumors had a slightly better outcome and this was irrespective of gender and HLA-type.

**Conclusions:** The prognostic traits of HLA-A*02 appear in this colon cancer cohort to act differently in male and female patients. Also CD8+ infiltration is different between genders. These findings suggest that men and women may have two different immune responses to malignancy (Table 1).

**Table 1:** Patient overview

Patient overview		N (%)	Women (%)	Men (%)
Cohort		520 (100)	249 (47.8)	271 (52.2)
Genus	Women	249 (47.8)	249 (100)	0 (0)
	Men	271 (52.2)	0 (0)	100 (0)
Localisation	Colon dx	241 (46.3)	117 (46.9)	124 (45.7)
	Transverse	47 (9)	23 (9.2)	24 (8.8)
	Colon sin	41 (8)	25 (19.1)	16 (5.9)
	Sigmoid	182 (35)	79 (31.7)	103 (38.1)
	Undetermined	9 (1.7)	5 (2.1)	4 (1.5)
Stage	II	230 (44.2)	108 (43.4)	122 (45.1)
	III	290 (55.8)	141 (56.6)	149 (54.9)
Treatment	Surgery	275 (52.9)	136 (54.6)	139 (51.2)
	Surgery + adj chemotherapy	245 (47.1)	113 (45.4)	132 (48.7)

##### 3 Role of Microvesicles in the transfer and in the transformation of melanoma cell lines

###### Giusy Gentilcore^1^, Selma Maacha^1^, Abbirami Shatappan^1^, Giuseppe Palmieri^2^, Paolo A. Ascierto^3^ and Jean-Charles Grivel^1^

####### ^1^Deep Phenotyping Core, Sidra Medicine, Doha, Qatar; ^2^Istituto Nazionale Tumori Fondazione G. Pascale, I-80131, Naples, Italy; ^3^Unit of Cancer Genetics, Institute of Biomolecular Chemistry, CNR, I-07100, Sassari, Italy

**Correspondence:** Giusy Gentilcore - ggentilcore@sidra.org

*Journal of Translational Medicine* 2020, **18(Supp 1)**:3

**Background:** Melanoma remains one of the most aggressive and heterogeneous skin cancer, which is often refractory to conventional chemotherapy. Nevertheless, it responds well to both immuno-and targeted therapy, which is focused on inhibiting the most common signaling pathway involved in melanoma transformation including the mitogen-activated protein kinase (MAPK) pathway. However, mechanisms of drug resistance have been described, some involving the release of extracellular vesicles (EVs). EVs are play an important role as intercellular communication mediators that can influence the phenotype and function of receiving cells. The aim of our study is to investigate the role of EVs in the mechanisms of drug resistance and phenotypic alteration in primary melanoma cell lines MEL50 BRAF-V600*mut* and M257 BRAF-*Wild Type*.

**Materials and methods:** In order to define phenotypic and functional differences between the two cell types, we characterized their surfaceome with a panel of 361-PE-conjugated antibodies specific for cell surface proteins. We compared the extracellular vesicles produced by both cell line, quantitatively and qualitatively by NTA and flow cytometry.

**Results:** We identified 49 markers expressed by more than 30% of MEL50 cells and 69 markers expressed by more than 30% of M257 cells. Among these markers, 10 are exclusively expressed by MEL50 and 36 are exclusively expressed by M257. Defining a distinctive surfaceome for both cell lines. We have also characterized the EVs produced by these cell lines and showed that MEL50 produces 3 times as much EVs than M257. These EVs are indistinguishable by Nanoparticle tracking analysis. Preliminary flow cytometric characterization of individual EVs did not show a significant difference in the expression of the classic EVs markers CD81, CD82, CD63 and CD9.

**Conclusions:** The characterization of the cancer cell surfaceome of two primary melanoma cell lines, one BRAF-V600*mut* and one BRAF-*Wild Type*, uncovered very distinctive phenotypes. While the expression of classic EVs markers was similar for EVs produced by either cell line, the extension of EVs marker characterization to the whole surfaceome of the parental cell line, may reveal the same heterogeneity, which could be used as biomarkers to identify BRAF mutated or wild type melanomas in liquid biopsies, and opens the door to investigating the role of specific EVs in drug resistance and phenotypic transformation.

## Immunotherapy Bridge 2019

### Trends in immunotherapy session

#### Oral communications

##### 4 Durvalumab induces an NK cell response associated with clinical benefit of patients with advanced NSCLC

###### Maria Libera Ascierto, Yashaswi Shrestha, Han Si, Chris Morehouse, Qu Zhang, Jixin Wang, Lydia Greenlees, Rebecca Halpin, Streicher, Rajiv Raja, Todd Hembrough

####### Translational Medicine, AstraZeneca, Gaithersburg, MD, 20878, USA

**Correspondence:** Maria Libera Ascierto - ml.ascierto@gmail.com

*Journal of Translational Medicine* 2020, **18(Supp 1)**:4

**Introduction:** The role of CD8 cells in determining clinical outcome to programmed death ligand-1 (PD-L1) blocking treatments has been well characterized, however, the contribution of NK cells is not well understood. This is partly due to the paucity of NK cell-specific markers that can identify NK cells in the tumor microenvironment (TME). We developed an NK cell-specific transcriptional signature to estimate the NK cell abundance in the TME. This signature, together with NK-chemokines shown to modulate the priming of adaptive immunity^1^ were investigated in patients with advanced non-small cell lung cancer (NSCLC) treated with a PD-L1 inhibitor, durvalumab.

**Methods:** Peripheral blood mononuclear cells (PBMCs) and Fluorescence-Activated Cell Sorted (FACS) NK/CD8 populations from three heathy donors were subjected to single cell RNA sequencing (scRNAseq, 10X Genomics) and transcriptome analysis (Affymetrix), respectively. Fresh frozen tumor biopsies from 97 NSCLC were profiled with RNA sequencing prior to durvalumab treatment; 29 of these had paired tumors procured 29 days following treatment with durvalumab. Kaplan–Meier (KM) analyses were performed to identify predictive effects of the NK cell-specific signature. Clinical trial: 1108/NCT01693562

**Results:** Transcripts over-expressed in sorted NK relative to CD8 cells were first identified (p < 0.01; fold > 3) and intersected with 28 mRNAs up-regulated in the NK cell cluster determined by scRNAseq, providing an 8 gene NK cell-specific transcriptional signature defined as MEDI-NK. MEDI-NK correlated with NK signatures recently described^2^, and included chemokines shown to induce an effective NK-response^1^. When evaluated in TCGA, higher expression of MEDI-NK was associated with good prognosis (Overall Survival, OS) of patients with melanoma and breast cancer (p value = 0.03 and = 0.001, respectively).

At baseline, MEDI-NK was highly correlated with the previously identified IFNγ signature^3^ and was associated with Progression Free Survival (PFS p value < 0.02) of NSCLC patients treated with durvalumab. Following treatment with durvalumab, the increased expression of MEDI-NK and of additional genes leading to NK-priming of adaptive immunity^1^ was observed to be associated with patients’ overall survival (OS p value < 0.01). Similar findings were not observed prior to durvalumab treatment.

**Conclusions:** Using single cell analysis, an NK cell-specific signature was developed to better define the role of NK cells in anti-PDL1 therapy. The increased expressions of the NK cell-specific gene signature and of genes leading to NK-cell priming of adaptive immune response were associated with clinical benefit to durvalumab.

**References**

1. Böttcher JP et al. Cell. 2018.
2. Barry KC et al. Nature Medicine. 2018.
3. Higgs B et al. Clinical Cancer Res. 2018.

##### 5 Ipilimumab and stereotactic radiosurgery in melanoma brain metastases: a retrospective monoistitutional experience

###### Valentina Borzillo^1^, Rossella Di Franco^1^, Fabrizio Cammarota^1^, Paolo A. Ascierto^2^, Antonio M. Grimaldi^2^, Ester Simeone^2^, Lucia Festino^2^, Vito Vanella^2^, Diana Giannarelli^3^, Paolo Muto^1^

####### ^1^Radiation Oncology Unit, Istituto Nazionale Tumori - IRCCS - Fondazione G. Pascale - Naples, Italy; ^2^Medical Oncology and Innovative Therapies, Istituto Nazionale Tumori - IRCCS - Fondazione G. Pascale - Naples, Italy; ^3^Statistical Unit, Regina Elena National Cancer Institute, Rome, Italy

**Correspondence:** Valentina Borzillo - v.borzillo@istitutotumori.na.it

*Journal of Translational Medicine* 2020, **18(Supp 1)**:5

**Background:** Ipilimumab (Ipi), an anti-cytotoxic T-lymphocyte-associated antigen 4 (CTLA-4) monoclonal antibody, has been shown to improve survival in patients (pts) with advanced melanoma [1–3]. Several retrospective studies have shown how the combination of radiotherapy (RT) and Ipi in the treatment of melanoma brain metastases (MBMs) pts improves the outcomes, without however clarifying the exact timing of the two modalities [3–10]. The purpose of this study is to evaluate overall survival (OS), local control (LC) (*in SRS field*) of the lesion treated, and intracranial control (IC) (*out SRS field*) in MBMs pts receiving Ipi and Stereotactic Radiotherapy (SRT)/Radiosurgery (SRS) performed with Cyberknife^®^ (CK) System.

**Materials and methods:** Since December 2012 until December 2018 we treated 63 (34 M and 29 F) MBMs pts, of these 53 received RT + Ipi and 10 RT alone (NO-IPI group). Patient and treatment characteristics were in Table 1. We divided the pts into 3 different groups based on therapies timing: 18 in RT PRE-IPI, 20 in RT CONCOMITANT (CONC) IPI, 15 in RT POST-IPI group. Ipi was administered intravenously at a dose of 3 mg/kg over 90 min every 3 weeks for 4 doses. A total of 127 lesions, were treated with SRS/SRT performed by CK. We evaluated the local response according to RECIST criteria. We assessed LC as the sum of complete response, partial response and stable disease, IC and median OS from the date of the SRS/SRT procedure.

**Table 1:** Patient and treatment characteristics

		NO IPI (10 pts)	RT POST IPI (15 pts)	RT CONC IPI (20 pts)	RT PRE IPI (18 pts)	TOTAL (63 pts)
Sex	M	7	5	9	13	34
	F	3	10	11	5	29
Age	Years					
	Median	64	62	55	63	60
	Range	40–77	29–81	28–80	32–80	28–81
ECOG PS	0	8	14	17	14	53
	1	2	1	3	4	10
RPA	Class I	0	0	1	3	4
	Class II	10	15	19	15	59
DS-GPA	1	0	0	0	0	0
	2	1	3	3	0	7
	3	4	6	7	10	27
	4	5	6	10	8	29
Melanoma site	Cutaneous	8	14	18	17	57
	Mucosal	1	1	1	0	3
	Unknown	1	0	0	1	2
	Ocular	0	0	1	0	1
Time between diagnosis and BMs	Months					
	Median	34	37	23	34	34
	Range	0–192	0–240	0–228	3–240	0–240
Extracranial disease	Yes	6	23	6	14	49
	No	5	1	1	7	14
LDH pre-RT	Normal	4	9	11	9	33
	High	4	5	7	6	22
	NA	2	1	2	3	8
BRAF status	Mutated	8	5	7	9	29
	Wild tipe	2	10	13	8	33
	NA				1	1
Neurological symptoms	Asymptomatic	5	15	14	14	48
	Symptomatic	5	0	6	4	15
Steroid treatment pre-RT	Yes	5	5	7	9	26
	No	4	8	13	9	34
	NA	1	2	–	–	3
Number of BMs treated		16	34	38	39	127
Lesion size	Median (mm)	9	9	8	8	8
	Range (mm)	2–30	3–36	2–42	3–37	2–42
	0–2 (cm)	13	28	33	31	105
	> 2–< 3 (cm)	3	3	3	7	16
	> 3 (cm)	0	2	2	1	5
	NA	–	1	–	–	1
Radiation Treatment	SRS (dose range 10–24 Gy)	11	20	21	21	75
	SRT (dose range 18–24 Gy)	4	4	6	10	24
Treatments before CK SRS/SRT	SRS	0	1	0	0	1
	WBRT	2	2	0	1	5
	Surgery	5	0	0	1	6
Treatments after CK SRS/SRT	SRS/SRT*	3#	7	7	8	25
	WBRT	0	4	4	7	15
	Surgery	1	0	0	1	2

**Results:** The median follow-up was 10.6 months (m) (range, 1.5–48.7 m). 59 pts for a total of 123 lesions were valuable for the follow-up. The median OS was 10.6 m (95% CI 8.5–12.7) for all pts, 10.7 m for IPI + RT and 3.3 m for NO IPI (p = 0.96). The median OS for single group was: 7.6 m for RT POST-IPI, 10.4 m for RT CONC IPI and 11.5 m for RT PRE-IPI (p = 0.89). The 1-year LC (*in SRS field*) was 53% for all lesions, 59% in IPI + RT and 8% in NO IPI (p = 0.001) (Fig. 1). The 1-year LC (*in SRS field*) for a single group was 74% for RT POST-IPI, 41% for RT CONC IPI and 48% for RT PRE-IPI groups (p = 0.002) (Fig. 2). The 1-year IC (*out SRS field*) was 45% for all pts, 44% for IPI + RT and 51% for NO IPI (p = 0.73). The 1- and 2-year OS of patients with LC was 50% and 25% vs 30% and 4% of patients without LC respectively (p = 0.02).

**Fig. 1:**
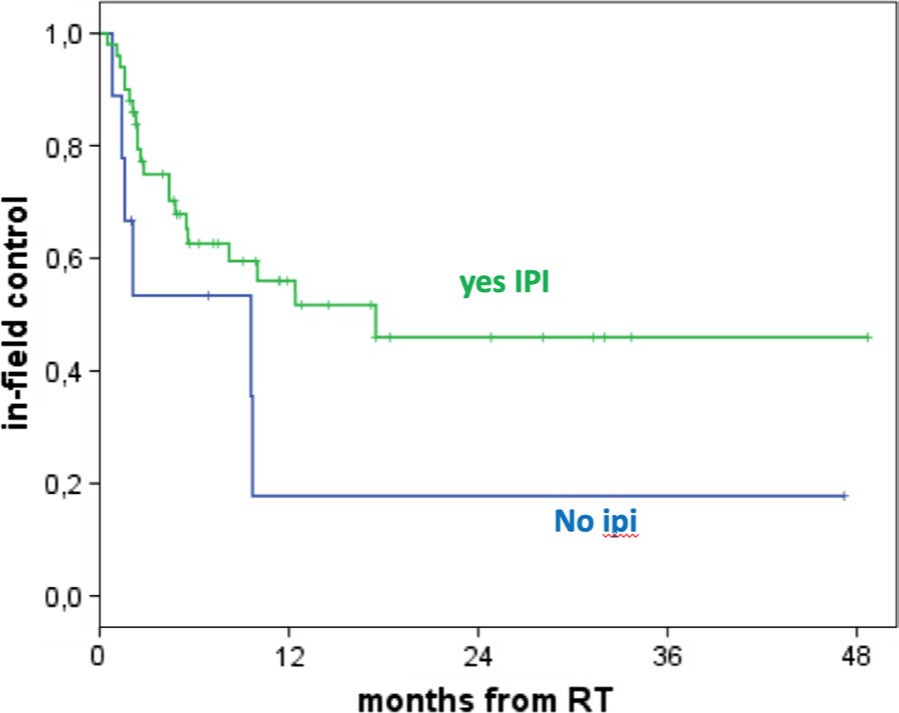
Local control according to ipi

**Fig. 2:**
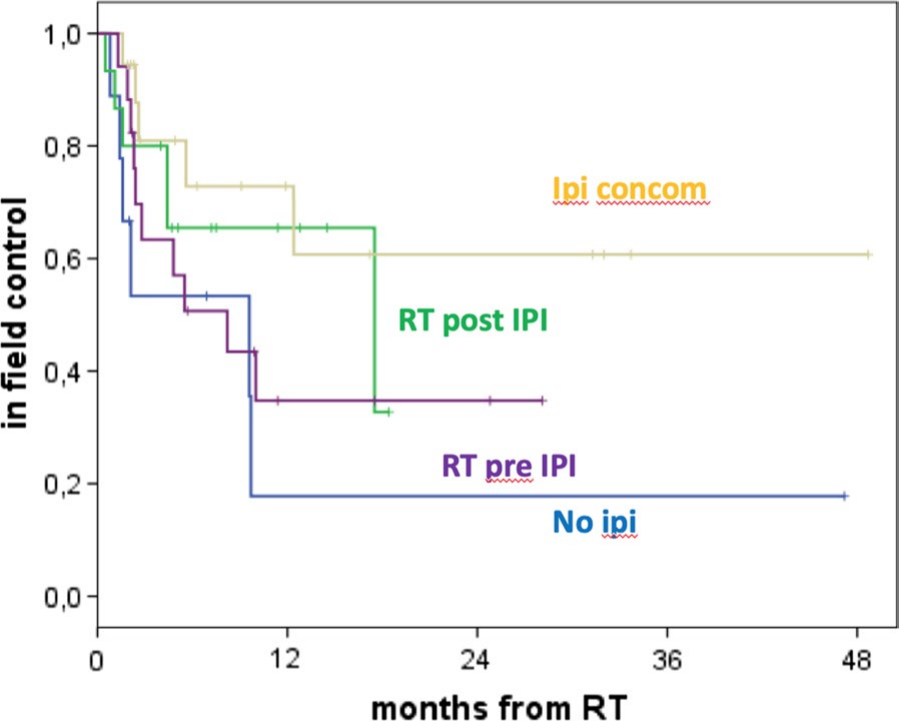
Local control according to the 4 groups

**Conclusions:** Our retrospective experience suggests that the combination of Ipi and SRS/SRT in MBMs pts can improve outcomes with a low toxicity profile. The optimal timing of combination Ipi and RT remains unclear, but from our experience it would seem to be a benefit on LC with SRS delivered after Ipi. The recruitment of a greater number of pts, a longer follow-up and new prospective studies are needed to demonstrate the role of Ipi in the treatment of MBMs and the better sequence with RT.

**References**

1. Hodi FS, O’Day SJ. Improved survival with ipilimumab in patients with metastatic melanoma. N Engl J Med. 2010; 363:711–23.
2. Lebbe C, McDermott DF. Ipilimumab improves survival in previously treated, advanced melanoma patients with poor prognostic factors: subgroup analyses from a phase III trial [abstract]. Ann Oncol. 2010;21(suppl 8):401.
3. Margolin K, Ernstoff MS. Ipilimumab in patients with melanoma and brain metastases: an open-label, phase 2 trial. Lancet Oncol. 2012;13(5):459–65.
4. Knisely JP, Yu JB. Radiosurgery for melanoma brain metastases in the ipilimumab era and the possibility of longer survival. J Neurosurg. 2012;117:227–33.
5. Schoenfeld JD, Mahadevan A, Ipilmumab and cranial radiation in metastatic melanoma patients: a case series and review. J Immunother Cancer. 2015;3:50
6. Patel KR1, Shoukat S. Ipilimumab and Stereotactic Radiosurgery Versus Stereotactic Radiosurgery Alone for Newly Diagnosed Melanoma Brain Metastases. Am J ClinOncol. 2015 May 16.
7. Kiess AP1, Wolchok JD2. Stereotactic radiosurgery for melanoma brain metastases in patients receiving ipilimumab: safety profile and efficacy of combined treatment. Int J Radiat Oncol Biol Phys. 2015;92(2):368–75.
8. Tazi K1, Hathaway A, Chiuzan C, Shirai K. Survival of melanoma patients with brain metastases treated with ipilimumab and stereotactic radiosurgery. CancerMed. 2015 Jan;4(1):1–6.
9. Silk AW1, Bassetti MF, West BT, Tsien CI, Lao CD. Ipilimumab and radiation therapy for melanoma brain metastases. Cancer Med. 2013;2(6):899–906.
10. Mathew M1, Tam M. Ipilimumab in melanoma with limited brain metastases treated with stereotactic radiosurgery. Melanoma Res. 2013;23(3):191–5.

##### 6 Radiation therapy exposes immunogenic mutations to the immune system in a breast cancer model

###### Claire Lhuillier^1*^, Nils Rudqvist^1^, Takahiro Yamazaki^1^, Lorenzo Galluzzi^1,2,3,4^, Sandra Demaria^1,2^

####### ^1^Department of Radiation Oncology, Weill Cornell Medicine, New York, NY, USA; ^2^Sandra and Edward Meyer Cancer Center, New York, NY, USA; ^3^Department of Dermatology, Yale School of Medicine, New Haven, CT, USA; ^4^Université Paris Descartes/Paris V, Paris, France

**Correspondence:** Claire Lhuillier - cfl2002@med.cornell.edu

*Journal of Translational Medicine* 2020, **18(Supp 1)**:6

**Background:** Growing evidence suggests that mutation-associated neoantigens drive responses to immune checkpoint blockade (ICB) in tumors with high mutational burden [1]. One factor that limits the recognition of these neoantigens by T cells is the level of expression of the mutated gene product in cancer cells. In the BALB/c-derived 4T1 mouse model of ICB-refractory metastatic breast cancer, we have previously shown that tumor-targeted radiation therapy (RT) combined with CTLA4 blockade induces CD8^+^ T cell-mediated regression of irradiated tumors and inhibits lung metastases [2]. Analysis of the T-cell receptor (TCR) repertoire indicated that unique clonotypes expand in treated tumors, suggesting that tumor rejection involves T cells reactive to a set of tumor antigens that are made available to the immune system by RT [3]. Therefore, we hypothesize that RT increases the expression of genes containing immunogenic mutations and hence promotes priming of neoantigen-specific T cells.

**Materials and methods:** We performed whole-exome sequencing and RNA sequencing of untreated and irradiated (8GyX3) 4T1 cells in vitro to identify tumor-specific neoantigens and determine which ones are upregulated by RT. These mutations were also documented in vivo, in 4T1 tumors harvested before and after treatment (8GyX3 + anti-CTLA4). Dedicated algorithms were used to predict MHC-I and MHC-II-binding epitopes from these mutated genes. Peptides with a predicted affinity < 500 nM were synthesized and tested in vitro for binding in a MHC stabilization assay. The best candidates were used to vaccinate BALB/c mice, followed by challenge with 4T1 cells to test for the induction of protective anti-tumor immunity.

**Results:** Out of 309 total mutations initially identified in 4T1 cancer cells, two MHC-I and one MHC-II neoepitopes were immunogenic in vaccination experiments as assessed by IFNγ/TNFα response after T cell re-stimulation. These neoepitopes were encoded by genes upregulated by RT. Vaccination with these three neoantigens induced a significant tumor growth delay in mice only when vaccination was combined with tumor-targeted RT. We observed significant changes in the intratumoral TCR repertoire in vaccinated mice. In addition, in vivo killing experiments demonstrated a potent cytolytic activity of T cells from vaccinated mice towards one of these neoepitopes. These results were confirmed in vitro after MHC-I blockade of the peptide-loaded target cells. Mass-spectrometry analyses of MHC-I-bound peptides are currently ongoing to assess the differences in presented antigens between untreated and irradiated cancer cells.

**Conclusions:** Overall, our data demonstrate the potential of RT to modulate the expression of antigenic mutations in tumors which could enhance responses to immunotherapy.

**References**

1. Schumacher TN, Schreiber RD. Neoantigens in cancer immunotherapy. Science. 2015;348(6230):69–74.
2. Demaria S, Kawashima N, Yang AM, Devitt ML, Babb JS, Allison JP, Formenti SC. Immune-mediated inhibition of metastases after treatment with local radiation and CTLA-4 blockade in a mouse model of breast cancer. Clinical cancer research: an official journal of the American Association for Cancer Research. 2005;11(2 Pt 1):728–34.
3. Rudqvist NP, Pilones KA, Lhuillier C, Wennerberg E, Sidhom JW, Emerson RO, Robins HS, Schneck J, Formenti SC, Demaria S: Radiotherapy and CTLA-4 blockade shape the TCR repertoire of tumor-infiltrating T cells. Cancer Immunology Research. 2018; 6(2):139–50.

## Immunotherapy Bridge 2019

### Drivers of immune responses session

#### Oral communications

##### 7 Toward transcriptomics-based prediction of response to Immune Checkpoint Inhibitor in patients with malignant melanoma

###### Domenico Mallardo^1^, Andrey Alexeyenko^2^, Mariaelena Capone^1^, Gabriele Madonna^1^, SuFey Ong^3^, Sarah Warren^3^, Kristina Viktorsson^4^, Bo Franzén^4^, Marilena Tuffanelli^1^, Marcello Curvietto^1^, Vito Vanella^1^, Lucia Festino^1^, Mariagrazia Vitale^1^, Giosuè Scognamiglio^5^, Joseph Beechem^3^, Gerardo Botti^5^, Alessandra Cesano^3^, Rolf Lewensohn^4^, Giuseppe V. Masucci^4^, Paolo Antonio Ascierto^1^

####### ^1^Dipartimento Melanoma, Immunoterapia Oncologica e Terapie Innovative, Istituto Nazionale Tumori IRCCS Fondazione “G. Pascale” Napoli, Italy; ^2^Department of Microbiology, Tumor and Cell Biology, Karolinska Institutet, Solna, Stockholm, Sweden. Science for Life Laboratory, Solna, Stockholm, Sweden; ^3^NanoString Technologies, Seattle, USA; ^4^Karolinska Institutet, Department of Oncology/Pathology, Karolinska University Hospital, Theme Cancer, Patient area head and neck, lung, and skin, Stockholm, Sweden; ^5^Dipartimento di Anatomia Patologica e Citopatologia, Istituto Nazionale Tumori IRCCS Fondazione “G. Pascale” Napoli, Italy

**Correspondence:** Paolo Antonio Ascierto - p.ascierto@istitutotumori.na.it

*Journal of Translational Medicine* 2020, **18(Supp 1)**:7

**Background:** The successful deployment of immune checkpoint inhibitors (ICI) in cancer immunotherapy relies on the responsiveness of an individual’s immune system for relief of that particular blockade in the cancer immunity cycle [1–3]. As most patients fail to respond to ICI, there is a need for biomarkers that can predict patient’s clinical benefit thereby identifying the patient population most likely to respond [4, 5]. The goal of this study was to augment the prediction accuracy by identifying and testing novel candidate biomarkers that could envisage response to ICI in patients with metastatic melanoma. The analysis had two specific features: validation against previously published predicting biomarkers and characterization of patients’ transcriptomes at individual gene and pathway levels, where network enrichment analysis (NEA) integrated disparate genes into pathway scores [6].

**Materials and methods:** Gene expression profiles were obtained using NanoString^®^ panels (IO 360 ™ beta or UIO) on formalin fixed paraffin embedded biopsies (FFPE) obtained from 30 stage IV metastatic melanoma patients treated with ipilimumab (anti-CTLA4) and 50 patients treated with Nivolumab (anti-PD1) of which 22 were first-line and 28 pretreated with ipilimumab. The samples originated from the pathological anatomy department of Istituto Nazionale Tumori IRCCS Fondazione “G. Pascale” of Napoli, Italy. All patients have appropriately signed informed consent. Statistical associations between treatment response and either gene or pathway score variables were estimated in linear models, which included covariates of known importance to ICI, such as mRNA expression of the checkpoint proteins and their ligands.

**Results:** First, candidate transcription-based biomarkers were discovered in our cohorts via correlation to clinical benefit and then analyzed for significance by covariate adjustment. Secondly, the candidates performance was validated using a similar previously published NanoString-based gene dataset [7]. In the ICI-naïve anti-PD1 cohort, we identified different genes which were informative on the clinical benefit regardless of the known determinants: F2RL1, ARG1 and ICAM5. In the anti-CTLA4 cohort, the individual gene analysis did not yield any significant and validated associations. However instead, we revealed a number of NEA-based correlates between “progression within 1 year” and pathways e.g. “Cell adhesion molecules”, “PECAM1 interactions”, as well as a number of immune-related differentially expressed gene lists.

**Conclusions:** NanoString-based transcriptomics and the cohort designs provided high-quality data for discovery of robust biomarkers of ICI response, holding promise for development of clinically useful diagnostic panels in malignant melanoma.

References

1. Cowey CL, Liu FX, Boyd M, Aguilar KM, Krepler C. Real-world treatment patterns and clinical outcomes among patients with advanced melanoma: A retrospective, community oncology-based cohort study (A STROBE-compliant article). Medicine (Baltimore). 2019 Jul; 98(28):e16328.
2. Balar AV, Weber JS. PD-1 and PD-L1 antibodies in cancer: current status and future directions. Cancer Immunol Immunother. 2017; 66(5):551–64.2.
3. [3] Robert C, Long GV, Brady B, Dutriaux C, Maio M, Mortier L et al. Nivolumab in previously untreated melanoma without BRAF mutation. N Engl J Med. 2015; 372(4):320–30.
4. Larkin J, Minor D, D’Angelo S Dutriaux C, Maio M, Mortier L et al. Overall survival in patients with advanced melanoma who received nivolumab versus investigator’s choice chemotherapy in CheckMate 037: a randomized, controlled, open-label phase III trial. J Clin Oncol. 2018; 36(4): 383–90.
5. Schachter J, Ribas A, Long GV, Arance A, Grob JJ, Mortier L et al. Pembrolizumab versus ipilimumab for advanced melanoma: final overall survival results of a multicentre, randomised, open-label phase 3 study (KEYNOTE-006). Lancet. 2017;390:1853–62.4.
6. Franco M, Jeggari A, Peuget S, Böttger F, Selivanova G, Alexeyenko A. Prediction of response to anti-cancer drugs becomes robust via network integration of molecular data. Sci Rep. 2019 Feb 20;9(1):2379.
7. Chen P-L, Roh W, Reuben A, Cooper ZA, Spencer CN, Prieto PA, et al. Analysis of immune signatures in longitudinal tumor samples yields insight into biomarkers of response and mechanisms of resistance to immune checkpoint blockade. Cancer Discov. 2016 Aug 1;6(8):827–37.

**Ethics approval:** The study was approved by the internal ethics board of the Istituto Nazionale Tumori IRCCS Fondazione “G. Pascale” of Napoli Italy, approval number of registry 17/17 OSS.

**Acknowledgements:** The study was supported by the Institutional Project “Ricerca Corrente” of Istituto Nazionale Tumori IRCCS Fondazione “G. Pascale” of Napoli, Italy.

##### 8 ASPH, a focal target in immuno-oncology for therapeutic cancer vaccine and cell therapy development

###### Ildiko Csiki^1^, Steve Fuller^1^, Michael Lebowitz^1^, Hossein Ghanbari^1^, John Celebi^1^

####### ^1^Sensei Biotherapeutics, Gaithersburg, MD 20879

**Correspondence:** Ildiko Csiki - icsiki@senseibio.com

*Journal of Translational Medicine* 2020, **18(Supp 1)**:8

**Background:** SNS-301 is a first-in-class therapeutic cancer vaccine candidate targeting human aspartyl (asparaginyl) β-hydroxylase (ASPH). ASPH is a highly tumor specific antigen that is differentially overexpressed in multiple human cancers but not in healthy adult tissue and is associated with tumor cell growth, motility and invasiveness. SNS-301 is engineered to express an ASPH fusion product within an inactivated λ-bacteriophage viral vector (phage display) to activate both innate and adaptive arms of the immune system. Extensive pre-clinical data demonstrated the immunotherapy’s ability to overcome tumor self-tolerance and provide anti-tumor immunostimulatory effect including strong activated, functional intra-tumoral CD8+ T cell infiltration.

**Materials and methods:** SNS-301 was tested in a phase I clinical trial via intradermal administration using a 3 M micro-needle injection system in ASPH overexpressing biochemically recurrent prostate cancer patients (pts). Twelve pts with detectable levels of ASPH received 3–23 doses of SNS-301.

**Results:** The immunotherapy was well tolerated with only 3 pts. experiencing an adverse event (AE) considered at least possibly related to study drug. All AEs were ≤ grade 3 and no dose-limiting toxicity was observed. All pts. experienced NK cell activation as well as dose-dependent ASPH-specific immune responses including CD4+ and CD8+ T-cell and B cell dependent immune responses. Anti-tumor activity and disease stabilization was observed in 8/12 pts. (67%) with declines noted in both overall PSA level and increases in PSA doubling rate.

**Conclusions:** SNS-301 is a novel immunotherapy that may overcome prior challenges of cancer vaccines and cell therapies. Based on the pre-clinical and phase I results, multiple phase II programs were initiated in ASPH positive patients across many tumor types to evaluate SNS-301 as an active product in the cancer-immunity cycle both as monotherapy and combination therapy with checkpoint inhibitors. A combination phase II study of SNS-301 with pembrolizumab in ASPH positive checkpoint resistant head and neck cancer patients is currently enrolling (NCT04034225). Additionally, ASPH is also in pre-clinical development as a cell therapy target in both heme and solid malignancies.

## Immunotherapy Bridge 2019

### Poster

#### 9 Is tumor mutational burden a prognostic marker in AJCC stage II melanoma?

##### Teresa Amaral^1,2^, Tobias Sinnberg^1^, Christopher Schroeder^3^, Elena Sofia Linder^3^, Heike Niessner^1^, Irina Bonzheim^4^, Thomas Eigentler^1^, Falko Fend^4^, Olaf Rieß^3^, Claus Garbe^1^

###### ^1^Center for Dermatooncology, Department of Dermatology, Eberhard Karls University of Tuebingen, Tuebingen, Germany; ^2^Portuguese Air Force, Health Care Direction, Lisbon, Portugal; ^3^Institute of Medical Genetics and Applied Genomics, University of Tuebingen, Calwerstr. 7, 72076, Tuebingen, Germany; ^4^Institute of Pathology and Neuropathology, Liebermeisterstr. 8, University Hospital Tuebingen, Tuebingen, Germany

####### **Correspondence:** Teresa Amaral - teresa.amaral@med.uni-tuebingen.de

*Journal of Translational Medicine* 2020, **18(Supp 1)**:9

**Background:** Tumor mutational burden (TMB) has been shown to be predictive of a good response to immunotherapy in stage IV melanoma and also other tumors, and is starting to be used as an inclusion criterion in ongoing clinical trials. However, the prognostic value of this marker is jet to be validated, also in earlier stages. We analyzed data from primary melanoma of stage II patients from the TCGA database and found that TMB could be prognostic in this collective. In this study, we intended to validate the prognostic value of TMB in a stage II cohort of melanoma patients from our department.

**Materials and methods:** We included patients with stage II melanoma diagnosed between 2000 and 2018 in the University Hospital of Tuebingen and for whom formalin-fixed and paraffine embedded normal and tumor tissue were available. Tumor and normal DNA sequencing was performed using a next generation sequencing (NGS) panel that covers 693 genes, 7 promotor regions and the intronic region of 26 genes with known fusion partners. TMB was expressed in mutations per megabase (mut/Mb) and the median TMB was used as cut-off to define high and low-TMB sub-groups. Descriptive analysis of patients’ characteristics and survival analysis were performed. The follow-up time was defined as the time between diagnosis and relapse or death.

**Results:** A total of 209 samples were included in the final analysis. More detailed information is presented in Table 1.

**Table 1:** Patients characteristics

	All n = 209	BRAFV600E/K n = 54	BRAFwt n = 136	BRAFOther n = 19	χ^2^
Age distribution					**0.056**
≤ 60	26%	40%	22%	20%	
61–75	35%	31%	37%	30%	
> 75	39%	29%	41%	50%	
Gender					0.913
Female	40%	61%	59%	35%	
Male	60%	39%	41%	65%	
Tumor localization					**0.002**
Trunk	28%	44%	21%	30%	
Lower extremity	32%	32%	36%	10%	
Upper extremity	12%	9%	14%	5%	
Head/neck	28%	15%	29%	55%	
Histological subtype					**< 0.0001**
SSM	37%	56%	32%	20%	
NM	24%	32%	19%	35%	
LMM	10%	0	10%	35%	
ALM	18%	11%	24%	0	
Others	11%	2%	15%	10%	
Tumor thickness					0.223
1.01–2.0 mm	14%	15%	15%	5%	
2.01–4.0 mm	53%	52%	50%	75%	
> 4 mm	33%	33%	35%	20%	
Ulceration					0.987
Yes	62%	61%	62%	65%	
No	38%	39%	38%	35%	
Regression					**0.001**
Yes	14%	18%	11%	20%	
No	70%	80%	65%	80%	
N/A	16%	2%	24%	0	
Stage at initial diagnosis					0.885
IIA	43%	44%	43%	40%	
IIB	33%	32%	33%	40%	
IIC	24%	24%	24%	20%	

The median TMB was slightly higher in the whole collective (median TMB = 14 mut/Mb) when compared to the subgroup of patients with BRAFV600E/K mutation (median TMB = 11 mut/Mb). The highest TMB was observed in patients with other BRAF mutations (median TMB = 55 mut/Mb).

When analyzing the whole collective, we found no difference in terms of median relapse-free survival (mRFS; p = 0.4689) and median overall survival (mOS; p = 0.5534) for patients with high and low-TMB. In patients harboring a BRAFV600E/K mutation the same results were observed, when the median TMB for this cohort was used as cut-off (mRFS; p = 0.3235 and mOS; p = 0.7547).

In the multivariate Cox Hazard analysis including gender, tumor localization, histological subtype, age, tumor thickness, ulceration and TMB as a continuous variable, only age and tumor thickness were significant (p < 0.0001 and p = 0.001, respectively). The p-value for TMB was 0.2.

**Conclusions:** Our analysis was unable to confirm the results from the TCGA database and TMB was not a prognostic marker in our cohort of stage II melanoma.

#### 10 Incidence and clinical implications of late immune-related adverse events in long responders to PD-1/PD-L1 checkpoint inhibitors: a multicenter study

##### Olga Nigro^1^, Graziella Pinotti^1^, Raffaele Giusti^2^, Marco Filetti^2^, Federica De Galitiis^3^, Francesca Romana Di Pietro^3^, Melissa Bersanelli^4^, Alessandro Lazzarin^4^, Annamaria Catino^5^, Pamela Pizzutillo^5^, Marco Russano^6^, Mariangela Torniai^7^, Biagio Ricciuti^8^, Alessandro Russo^9^, Marianna Tudini^10^, Elena Bolzacchini^11^, Pietro Di Marino^12^, Erika Rijavec^13^, Ilaria Vallini^14^, Corrado Ficorella^15,16^, Alessio Cortellini^15,16^

###### ^1^Medical Oncology, ASST Sette Laghi, Ospedale di Circolo e Fondazione Macchi, Varese, Italy; ^2^Medical Oncology, Azienda Ospedaliero-Universitaria Sant’Andrea, Roma, Italy; ^3^Oncologia ed Oncologia Dermatologica, IDI-IRCCS, Roma, Italy; ^4^Medical Oncology, Azienda Ospedaliero-Universitaria di Parma, Italy; ^5^Thoracic Oncology Unit, Clinical Cancer Centre “Giovanni Paolo II”, Bari, Italy; ^6^Medical oncology, Campus Bio-Medico, Roma, Italy; ^7^Medical Oncology, Azienda ospedaliera universitaria Ospedali Riuniti di Ancona, Italy; ^8^Medical Oncology, Ospedale Santa Maria della Misericordia, Perugia, Italy; ^9^Medical Oncology, A.O. Papardo di Messina, Italy; ^10^Medical Oncology, ASUR Marche, Area Vasta 2, Fabriano, Italy; ^11^Medical Oncology, ASST Lariana, Ospedale Sant’Anna, Como, Italy; ^12^Clinical Oncology Unit, S.S. Annunziata Hospital, Chieti, Italy; ^13^Medical Oncology, Policlinico di Milano, Milano, Italy; ^14^Study Coordinator, Medical Oncology, ASST Sette Laghi, Ospedale di Circolo e Fondazione Macchi, Varese, Italy; ^15^Medical Oncology, St. Salvatore Hospital, L’Aquila, Italy; ^16^Department of Biotechnology and Applied Clinical Sciences, University of L’Aquila, L’Aquila, Italy

####### **Correspondence:** Alessio Cortellini - alessiocortellini@gmail.com, corrado.ficorella@univaq.it

*Journal of Translational Medicine* 2020, **18(Supp 1)**:10

**Background:** Immunotherapy has become standard of care for an increasing number of tumors. Patients exposed to these drugs have a chance of developing immune-related adverse events (irAEs). In general, irAEs occur quite early, mostly within weeks to 3 months after initiation of immune checkpoint blockers. Being treatments relatively innovative, “late” irAEs are still unknown.

**Methods:** This is a multicenter retrospective study of advanced cancer patients (any histology, regardless of treatment line) treated with anti-PD-1/PD-L1 (mono)immunotherapy, with a minimum time to treatment failure (TTF) of 12 months. IrAEs were categorized into “early” (which occurred within the first 12 months of treatment) and “late”. An explorative analysis of clinical outcomes (TTF and Overall Survival—OS) was performed. The data cut-off analysis was August 2019.

**Results:** We evaluated 318 consecutive patients; the commencement date ranged from September 2013 to August 2018. Median age was 68.6 years (32–90); patients characteristics are summarized in Table 1. 175 patients (55.5%) experienced any grade early-irAEs, while 110 (34.6%) experienced any grade late-irAEs (p = 0.0013); 13 patients (4.1%) experienced G3/G4 early-irAEs, while 12 (3.8%) G3/G4 late-irAEs (p = 0.8446). There was a significant association between the occurrence of any grade early-irAEs and late-irAEs (p = 0.0452), as well as between G3/G4 early-irAEs and late-irAEs (p = 0.0251). Table 2 summarized the irAEs occurrence according to the system/organ involved. Among patients who experienced early-irAEs, 63 (36%) experienced “multiple-site” irAEs (multiple sites/organs), while 17 patients (15.4%) experienced multiple-site late-irAEs (p = 0.0040). Table 3 summarized the clinical management of early- and late-irAEs. The median period of follow-up was 22.2 months. The median time to irAEs onset were 3.1 and 16.1 months for early- and late-irAEs, respectively. Late irAEs were not significantly related to TTF (Fig. 1A), on the other hand, were significantly related to a prolonged OS (Fig. 1B). When adjusted for primary tumor (Table 4), late-irAEs were confirmed to be significantly related to a prolonged OS (HR = 0.25 [95% CI 0.11–0.55]; p = 0.0006).

**Table 1:** Patients characteristics. NSCLC (Non Small Cell Lung Cancer); SD (Stability of Disease); PR (Partial Response); CR (Complete Response)

	Patients N = 318 (%)
Age (years)	
Median (range)	68.6 (32–90)
Gender	
M	223 (70.13)
F	95 (29.87)
Histology	
NSCLC	200 (62.89)
Melanoma	87 (27.36)
Kidney	23 (7.23)
Others	8 (2.52)
ECOG PS	
0	199 (62.58)
1	102 (32.07)
≥ 2	17 (5.35)
Number of metastatic sites	
< 3	214 (67.3)
≥ 3	104 (32.7)
Metastasis	
CNS	40 (12.58)
Bone	60 (18.87)
Liver	28 (8.81)
Previous lines of treatments	
0	122 (38.37)
1	126 (39.62)
≥ 2	70 (22.01)
Immunotherapeutic agents	
Pembrolizumab	126 (39.62)
Nivolumab	187 (58.81)
Atezolizumab	5 (1.57)
Duration of Immunotherapy	
≥ 12 months < 18	121 (38.05)
≥ 18 months < 24	94 (29.56)
≥ 24 months < 36	80 (25.16)
≥ 36 months	23 (7.23)
Best response	
SD	88 (27.67)
PR	197 (61.95)
CR	33 (10.38)

**Table 2:** irAEs occurrence according to the system/organ involved

Any grade irAEs	Early-irAEs (patients-%)	Late-irAEs (patients-%)	P value
Overall population	175 (55.0)	110 (34.6)	0.0013
Skin	68 (38.9)	45 (40.9)	0.8212
Endocrine	49 (28.0)	17 (15.4)	0.0508
Gastrointestinal	39 (22.3)	15 (13.6)	0.1314
Pneumological	12 (6.9)	7 (6.4)	0.8792
Haepatic	8 (4.6)	3 (2.7)	0.5411
Rheumatologic	37 (21.1)	26 (23.6)	0.6943
Neurologic	1 (0.6)	7 (6.4)	0.0076
Others	47 (26.9)	11 (10.0)	0.0044
G3/G4 irAEs	13 (4.1)	12 (3.8)	0.8446

**Table 3:** Clinical management of early- and late-irAEs

	Early-irAEs (patients-%)	Late-irAEs (patients-%)	P value
Any grade irAEs	**175**	**110**	
Single-site irAEs	112 (64.0)	93 (84.5)	0.1339
Multiple-site irAEs	63 (36.0)	17 (15.4)	0.0040
Management			
No intervention (only supportive)	87 (49.7)	55 (50.0)	0.9783
Corticosteroids without discontinuation	69 (39.4)	38 (34.5)	0.5754
Corticosteroids with temporary discontinuation	19 (10.9)	6 (5.5)	0.1488
Corticosteroids with permanent discontinuation	–	11 (10)	0.0001

**Fig. 1:**
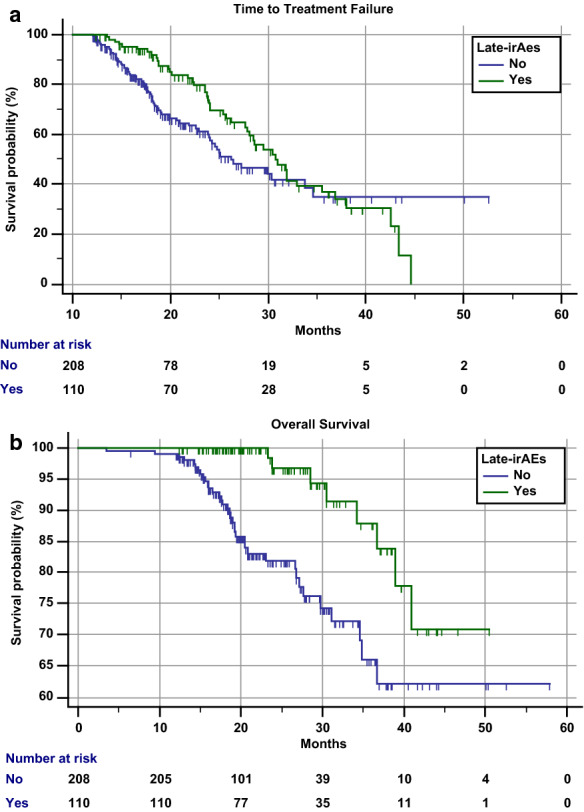
Kaplan–Meier survival curves according to the occurrence of late-irAEs (A) Time to Treatment Failure (B) Overall Survival

**Table 4:** Univariate and multivariate analysis for overall survival

Variable	Overall survival	
	Univariate analysis	Multivariate analysis
	HR (95% CI); *p value*	HR (95% CI); *p value*
Late-irAEs (any grade) Yes vs No	0.28 (0.13–0.62); *p *= *0.0015*	0.25 (0.11–0.55); *p *= *0.0006*
Primary tumor (NSCLC vs)		
Melanoma	0.43 (0.20–0.95); *p *= *0.0363*	0.39 (0.18–0.86); *p *= *0.0207*
Kidney	0.62 (0.19–2.05); *p *= *0.4366*	0.39 (0.12–1.33); *p *= *0.1340*
Others	1.01 (0.13–7.48); *p *= *0.9897*	0.65 (0.08–4.88); *p *= *0.6811*
Sex Male vs Female	0.94 (0.18–3.07); *p *= *0.6837*	–
Age (continuous)	1.02 (0.98–1.05); *p *= *0.2110*	–
Treatment line Non-first vs first	2.11 (0.94–4.82); *p *= *0.0688*	–
ECOG PS ≥ 2 vs 0–1	0.74 (0.18–3.07); *p *= *0.6837*	–
N of metastatic sites ≥ 3 vs < 3	1.05 (0.56–1.95); *p *= *0.8631*	–

**Conclusions:** Late-irAEs among long responders seem to have a mild/moderate incidence. They are mostly non-serious and clinical manageable, with a low rate of treatment discontinuation. In this positive-selected population, the occurrence of any grade late-irAEs seems to be furtherly related to a prolonged OS.

**Keywords:** immunotherapy; immune checkpoint; nivolumab; pembrolizumab; atezolizumab; immune-related adverse events.

- In the overall population median TTF was 28.6 months (95% CI 25.7–31.8; 122 events). In patients who experienced any grade late-irAEs median TTF was 30.8 months (95% CI 27.8–35.5; 47 events), while in patients who did not was 26.2 months (95% CI 23.9–33.8; 75 events) (HR = 0.72 [95% CI 0.54–1.04], p = 0.0794)

In the overall population median OS was Not Reached (274 censored). In both the patients who experienced (102 censored patients) and did not experienced any grade late-irAEs (172 censored patients) the median OS was Not Reached (p = 0)

#### 11 Immune-related adverse events (IrAEs) in patients receiving immune checkpoint inhibitors

##### Alessia Ferrarini^1^, Devis Benfaremo^1^, Giulia Rossetti^1^, Francesca Morgese^2^, Giovanni Pomponio^1^, Rossana Berardi^2^, Armando Gabrielli^1^

###### ^1^Clinica Medica, Dipartimento di Scienze Cliniche e Molecolari, Università Politecnica delle Marche, Ancona, Italy; ^2^Clinica Oncologica, Dipartimento di Scienze Cliniche e Molecolari, Università Politecnica delle Marche, Ancona, Italy

####### **Correspondence:** Alessia Ferrarini - alessiaferrarini1@gmail.com

*Journal of Translational Medicine* 2020, **18(Supp 1)**:11

**Background:** Recent introduction of anti-PD-1 (Nivolumab and Pembrolizumab) and anti-PD-L1 (Atezolizumab, Darvalumab) immune checkpoint inhibitors revolutionized oncological guidelines. IrAEs reported in clinical trials account to a maximum of 85%, while grade 3/4 of toxicity were reported in 10% of patients. Quality of AEs reporting in RCTs is satisfactory, but methods for data collection and analysis are unclear. The purpose of the study is to establish a cohort of cancer patients treated with immune checkpoint inhibitors (PD-1/PD-L1 inhibitors) in order to determine incidence and characteristics of irAEs in a real-world setting and improve clinical management.

**Materials and methods:** We conducted a prospective cohort study in patients receiving anti-PD-1/PDL1 drugs for treatment of metastatic or locally advanced non-small cell lung cancer, renal cell carcinoma, squamous cell carcinoma of the head and neck, Hodgkin lymphoma starting from Jan 2019. We created a clinical pathway aimed to improve management of patients at risk for IRAEs. In particular, definite recommendations have been implemented for cases fulfilling criteria for suspected irAEs. They concern procedures for evaluation and diagnosis, specific treatments and rules for drug discontinuation. IrAEs have been defined and graded according to Common Terminology Criteria for Adverse Events vs 5.0. Management strategies have been adapted by a multidisciplinary panel, basing on the ASCO guidelines, which represent current best clinical practice.

**Results:** Thirty-seven patients (F/M: 12/25, aged 69, range 38–92) have been enrolled. They were observed at baseline visit, and at weeks 4, 8, 12. Eleven patients had melanoma, seven had renal cell carcinoma, seventeen Non-small-cell lung carcinoma, one had Hodgkin lymphoma and one head and neck cancer. During the observation period, eight patients developed irAEs (21%) (three under treatment with Nivolumab, three with Pembrolizumab, one with Atezolizumab and one with Darvalumab). We observed different grade of severity: G1 in two patients that developed hepatitis and hypothyroidism, G2 in three patients that developed III-V-VII cranial nerve palsy and two PMR-like. In three patients (37.5%) irAEs were severe (G3): bullous dermatitis, interstitial pneumonia and myositis. No case of G4 were observed. Median time of insurgence of irAEs was 4.5 weeks. Twenty-nine (78%) are still under treatment. Five patients stopped anti-neoplastic therapy: three due to irAEs (G2–3), two for radiological or clinical progression. Three patients died.

**Conclusions:** Innovative tools are required in order to manage irAEs, prevent their potential relapse and to avoid useless interruption of therapy, with the goal to improve patients outcome.

**References**

1. Arnaud-Coffin P. A systematic review of adverse events in randomized trials assessing immune checkpoint inhibitors. Int J Cancer. 2019 Aug 1.
2. Champiat S, Lambotte O, Barreau E, et al. Management of immune checkpoint blockade dysimmune toxicities: a collaborative position paper. Annals of Oncology 2016;27: 559–74.

#### 12 Immunoresponse by using flow cytometric High-dimensional analysis: new approach in pediatric Acute Lymphoblastic Leukemia as model for other type of cancers

##### Giusy Gentilcore^1^, Chiara Cugno^1^ and Jean-Charles Grivel^2^

###### ^1^Advanced Cell Therapy, Sidra Medicine, Doha; ^2^Deep Phenotyping Core, Sidra Medicine, Doha

####### **Correspondence:** Giusy Gentilcore - ggentilcore@sidra.org

*Journal of Translational Medicine* 2020, **18(Supp 1)**:12

**Background:** Acute Lymphoblastic Leukemia (ALL) patients is the most common malignancy in children and represents 75–80% of leukemia cases. The most frequent immunophenotype is B-cell precursor ALL (B-ALL) in which, signaling via the B cell receptor (BCR) and its precursor (pre-BCR), play a crucial role in tumor promotion. It has been reported that Leukemias originate from cells with stem characteristics (LSC) well described in Acute Myeloid Leukemia (AML) but controversial in ALL. We propose to identify these cells by their dysregulated signaling pathway, using a combination of phosphoflow (SNCP) and cell surface markers in a high dimension flowcytometric approach in pediatric ALL.

**Methods:** We enrolled a cohort of 20 B-ALL pediatric patients and adult healthy donors (HD) for a pilot study in order to set up the Single SCNP method. To evaluate the activation of ERK and STAT signal pathways, in addition to the phosphoprotein activation markers we developed a high-dimensional multicolor panel of 20 extracellular markers and applied it to 5 HD and 1 blood samples at baseline and after stimulation with Phorbol Myristate Acetate (PMA). The Spectraviewer Cytometer Aurora has been used to perform the experiments and the data have been analyzed with Cytobank using visualization tools like SPADE and viSNE algorithms.

**Results:** In this pilot study, we show that it is possible to perform high dimension phenotypic and functional panels using fluorescently labeled antibodies, and that this constitutes a major advantage for the study of pediatric samples where sample-size is limiting. By defining SPADE trees clustered on cell surface markers, we traced multiple phosphorylation events monitored with ERK1,2 (pT202/pY204), p38MAPK (pT180/pY182), STAT1 (pY701), STAT3 (pY705) and STAT5 (pY694) in HD and B-ALL sample at basal levels and after stimulation.

**Conclusions:** This study shows that this approach to characterize the activation pathways in different leukemia subpopulations, is feasible and potentially powerful enough to identify LSC. It can also be used as model for cancer patients were the sample size, as like pediatric samples, is very limited.

#### 13 Total RNA-transcriptomics for identification of predictors of overall survival in metastatic melanoma patients treated with anti-PD-1

##### Qingyang Xiao, Javier Oliver, Juan Luis Onieva, Pilar Piñeiro, Alicia Garrido-Aranda, Aurora Laborda-Illanes, Elena Gallego, Cynthia Robles-Podadera, Rosario Chica-Parrado, Daniel Prieto, Alfonso Sánchez, Vanessa De Luque, María José Lozano, Martina Álvarez, Pedro Jiménez, Emilio Alba, Miguel Berciano-Guerrero, Manuel Cobo, Isabel Barragán

###### Cancer Molecular Biology Laboratory. Biomedical Research Institute of Malaga (IBIMA), University Hospital of Malaga (Virgen de la Victoria), Centro de Investigaciones Médico-Sanitarias (CIMES)

####### **Correspondence:** Isabel Barragan - isabel.barragan.mallofret@gmail.com

*Journal of Translational Medicine* 2020, **18(Supp 1)**:13

**Background:** Immune Checkpoint Blockade (ICB) achieves up to 45% of response in advanced non-small-cell lung cancer and melanoma. However, its use is suboptimal because the resistance mechanisms are not defined and we lack good predictive biomarkers. This study aims at identifying functional biomarkers of response to anti-PD-1 treatment.

**Methods:** A retrospective pilot cohort of 16 patients with metastatic cutaneous melanoma treated with Nivolumab was categorized into extreme good or bad responders according to best response and treatment duration. Total RNA from FFPE tumor tissues was subjected to transcriptomics profiling by RNA-seq with ribosomal RNA depletion. Differential expression was calculated with DeSeq2, and pathway analysis with GSEA. Survival analysis was performed using Kaplan–Meier method.

**Results:** We have identified 140 genes as differentially expressed (DE) (adj p < 0.05) in good responders to Nivolumab. Interestingly, the genes are in their majority expressed in immune cells, in particular in the B cell lineage. GSEA shows mainly processes related to immune response, with a high B cells involvement. In addition, 22 genes are associated with improved overall survival, among which there are several genes coding for specific regions of both variable and constant domains of immunoglobulin chains, and the tumor gene *LGR5*, which is a cancer stem cells marker and is correlated with chemotherapy resistance in gastric cancer.

**Conclusion:** This is the first study reporting a total-ARN profiling of patients treated with ICB. It reveals a comprehensive signature of immune-cells specific genes that delineate the response. The overrepresentation of B cell lineage genes suggests unprecedented hypotheses for the response mechanisms.

## Melanoma Bridge 2019

### Melanoma as a model system session

#### Oral communications

##### 14 36 months and 18 months relapse-free survival (RFS) after (neo)adjuvant ipilimumab (IPI) + nivolumab (NIVO) in macroscopic stage III melanoma (OpACIN and OpACIN-neo trial)

###### Elisa A Rozeman^1^, Alexander M Menzies^2^, Judith M Versluis^1^, Irene LM Reijers^1^, Oscar Krijgsman^3^, Esmée P Hoefsmit^3^, Bart A van de Wiel^4^, Karolina Sikorska^5^, Trieu M Van^3^, Hanna Eriksson^6^, Carolien Bierman^4^, Petros Dimitriados^3^, Maria Gonzalez^2^, Kerwin Shannon^7^, Annegien Broeks^4^, Ron Kerkhoven^8^, Andrew J Spillane^7^, Winan van Houdt^9^, Robyn PM Saw^7^, Alexander CJ van Akkooi^9^, Richard A Scolyer^10^, Johan Hansson^6^, Ton NM Schumacher^3^, Georgina V Long^2^ and Christian U Blank^1,3^

####### ^1^Medical Oncology Department, Netherlands Cancer Institute/Antoni van Leeuwenhoek hospital (NKI-AVL), Amsterdam, Netherlands; ^2^Department of Medical Oncology, Melanoma Institute Australia, University of Sydney, North Sydney, NSW, Australia; ^3^Division of Molecular Oncology and Immunology, Netherlands Cancer Institute, Amsterdam, Netherlands; ^4^Department of Pathology, Netherlands Cancer Institute/Antoni van Leeuwenhoek hospital (NKI-AVL), Amsterdam, Netherlands; ^5^Department of Biometrics, Netherlands Cancer Institute/Antoni van Leeuwenhoek hospital (NKI-AVL), Amsterdam, Netherlands; ^6^Department of Medical Oncology, Karolinska Institutet, Stockholm, Sweden; ^7^Surgical Oncology, Melanoma Institute Australia, University of Sydney, North Sydney, Australia; ^8^Genomics Core Facility, Netherlands Cancer Institute/Antoni van Leeuwenhoek hospital (NKI-AVL), Amsterdam, Netherlands; ^9^Surgical oncology, The Netherlands Cancer Institute Antoni van Leeuwenhoek Hospital, Amsterdam, Netherlands; ^10^Department of Pathology, Melanoma Institute Australia, University of Sydney, North Sydney, Australia

**Correspondence:** Christian U Blank - c.blank@nki.nl

*Journal of Translational Medicine* 2020, **18(Supp 1)**:14

**Background:** Outcome of high-risk stage III melanoma patients was poor with a 5-year overall survival rate of < 50%. Adjuvant IPI improved 5-year RFS and OS, and adjuvant anti-PD-1 improved RFS further. Preclinical data suggested that neoadjuvant treatment might be more favorable due to a broader immune activation. The investigator-initiated OpACIN trial compared neoadjuvant with adjuvant IPI + NIVO, while the subsequent OpACIN-neo trial tested three different dosing schedules of neoadjuvant IPI + NIVO without adjuvant therapy. Concomitant neoadjuvant IPI + NIVO induced a high pathologic response rates of 77–80% [1, 2]. Here we present the 36- and 18 months RFS of the OpACIN and OpACIN-neo trial respectively.

**Methods:** In the phase 1b feasibility OpACIN trial, 20 stage IIIB/IIIC melanoma pts with palpable nodal disease were included. Pts were randomized to receive IPI 3 mg/kg plus NIVO 1 mg/kg, either adjuvant 4 courses, or split 2 courses neoadjuvant and 2 adjuvant. In the subsequent OpACIN-neo trial 86 pts were randomized to arm A: 2× IPI 3 mg/kg + NIVO 1 mg/kg Q3W (n = 30); arm B: 2× IPI 1 mg/kg + NIVO 3 mg/kg Q3W (n = 30); and arm C: 2× IPI 3 mg/kg Q3W followed immediately by 2× NIVO 3 mg/kg Q2W (n = 26). Pathologic response was defined as < 50% viable tumor cells and was centrally reviewed by a blinded pathologist. Landmark RFS rates were estimated using Kaplan–Meier method.

**Results:** After a median FU of 36.7 and 17.7 months only one of the of the 71 pts (1.4%) with a centrally confirmed pathologic response on neoadjuvant therapy had relapsed, while 15/23 (65.2%) of pathologic non-responders had relapsed. The estimated 3-year RFS rate was 80% (95% CI 59–100) for the neoadjuvant arm and 60% for the adjuvant arm (95% CI 36–100) (OpACIN trial). After a median follow-up of 17.7 months, median RFS was not reached in any of the arms from OpACIN-neo. Estimated 18-months RFS was 85% for all pts (95% CI 78%–93%), 90% for arm A (95% CI 80%–100%), 82% for arm B (95% CI 70%–98%) and 83% for arm C (95% CI 70%–100%). Translational analyses indicate that baseline tumor mutational burden and interferon-y gene expression score are synergistic predictors of response.

**Conclusions:** While OpACIN showed for the first time a potential benefit of neoadjuvant versus adjuvant immunotherapy, OpACIN-neo confirmed the high pathologic response rates that can be achieved by neoadjuvant IPI + NIVO. Both trials indicate that pathologic response is an excellent surrogate marker for relapse free survival.

**Clinical trial information:** NCT02437279, NCT02977052

References

1. Rozeman EA, Menzies AM, van Akkooi ACJ et al. Identification of the optimal combination dosing schedule of neoadjuvant ipilimumab plus nivolumab in macroscopic stage III melanoma (OpACIN-neo): a multicentre, phase 2, randomised, controlled trial. Lancet Oncol. 2019; 20:948–60.
2. Blank CU, Rozeman EA, Fanchi LF et al. Neoadjuvant versus adjuvant ipilimumab plus nivolumab in macroscopic stage III melanoma. Nat Med. 2018; 24:1655–61.

##### 15 Preliminary results of a Neoadjuvant combo-immunotherapy with ipilimumab and nivolumab in locally advanced or limited metastatic melanoma

###### Pier Francesco Ferrucci^1^, Laura Pala^1^, Fabio Conforti^1^, Luigi Nezi^2^, Teresa Manzo^2^, Emilia Cocorocchio^1^

####### Melanoma Unit^1^ and Department of Experimental Oncology^2^, European Institute of Oncology, Milan, Italy

**Correspondence:** Pier Francesco Ferrucci - pier.ferrucci@ieo.it

*Journal of Translational Medicine* 2020, **18(Supp 1)**:15

**Background:** Unprecedented advances have been reached in the treatment of Melanoma using immune checkpoint inhibition, thanks to a better understanding of the molecular basis of tumor development and its interaction with the host.

Anticipating treatment with neoadjuvant therapy has the potential to significantly improve the clinical outcome of patients with locally/regionally advanced melanoma having the advantage to allow the assessment of initial tumor response and to be probably more efficient/better tolerated, due to the lower tumor burden and the enhanced amount of neoantigens triggering the TCR in the presence of disease.

In order to increase our knowledge in the field of drug resistance and/or response biomarkers, another great advantage of neoadjuvant trials is the availability of samples before and after systemic therapy for conducting novel mechanistic and biomarker studies in the circulation and the tumor microenvironment.

**Materials and methods:** Thirty-five stage III B-D oligometastatic stage IV melanoma patients will be screened and treated with neoadjuvant therapy with Ipilimumab 1 mg/kg + Nivolumab 3 mg/kg every 3 weeks for 4 cycles, will receive surgery and then an adjuvant therapy with Nivolumab 480 mg every 4 weeks for 6 cycles.

Sample collection (tissue, blood, urine and feces) for diagnosis, biomarker and molecular analysis will be collected at baseline, after each cycle (except tissue) surgery and afterwards in the adjuvant setting.

**Results:** Proteomic analysis of sera of treated patients, with particular emphasis on cytokines and chemokines, are being performed in order to identify possible markers associated with a better clinical outcome. The antitumor immune response in peripheral blood lymphocytes has been monitored, in order to evaluate whether the combination of antiCTLA4 and anti-PD1 is able to increase the number and/or the repertoire of melanoma-specific T-cells after treatments.

Gene sequencing analysis and expression profiling of genes involved in immune response by different means will also be evaluated in order to detect possible variations induced by the treatment on a molecular level. Finally, data on the modification induced by the disease and treatment on the microbioma and microbiota at different time points, showed interesting influences in maintaining or creating a beneficial equilibrium.

All these preliminary data will be presented and discussed together with efficacy/toxicity, based on percentages of pathological complete responses reached at surgery.

**Conclusion:** Understanding the molecular mechanisms of metastatic spread and exploiting such knowledge in prevention will likely have a profound impact on melanoma prognosis in advanced stages.

In a melanoma patient’s population including stage IIIB-C, or IV with potentially resectable disease, neoadjuvant immunotherapy was feasible, while identification of biomarkers of response and prognosis is ongoing in order to allow a better patient’s selection.

## Melanoma Bridge 2019

### Mechanism of resistance and drivers of response session

#### Oral communications

##### 16 Primary resistance to immune-checkpoint inhibitors in patients with metastatic melanoma

###### Teresa Amaral^1,2^, Zeinab Assi^1^, Ulrike Keim^1^, Andreas Meiwes^1^, Ioannis Thomas^1^, Julia Wilhelmi^1^, Thomas Eigentler^1^, Claus Garbe^1^, Olivia Seeber^1^

####### ^1^Centre for Dermatooncology, Department of Dermatology, Eberhard Karls University, Tuebingen, Germany; ^2^Portuguese Air Force Health Care Direction, Lisbon, Portugal

**Correspondence:** Teresa Amaral - teresa.amaral@med.uni-tuebingen.de

*Journal of Translational Medicine* 2020, **18(Supp 1)**:16

**Background:** The approval of immunotherapy and targeted therapy have changed the treatment landscape of stage IV melanoma. Nevertheless, there are still patients that do not derive benefit from these therapies, particularly when primary resistance is present.

**Materials and methods:** Here we analyzed patients diagnosed with stage IV melanoma between January 2015 and December 2018, and treated with first-line immunotherapy. Primary resistance was defined as disease progression at the time of first radiologic evaluation, after immunotherapy start. Patients with stable disease, partial response or complete response were considered to have disease control (DC). Follow-up time was defined as the time between stage IV diagnosis and dead or last contact. Descriptive analysis of patients’ characteristics and prognostic factors was performed. Progression-free survival (PFS), 1, 2 and 3-y survival and overall survival (OS) were also analyzed.

**Results:** A total of 530 patients with stage IV melanoma were analyzed; 347 patients received first-line immunotherapy and 144 patients were considered primary resistant. More information about patients’ characteristics can be found in Table 1. The median follow-up was 23 months (95% CI 20.5–25.5).

**Table 1:** Patients’ characteristics of the whole collective

Characteristics	All n = 530	IT collective n = 347*	IT n = 347*		$\varvec{\chi}$^2^ test♣
			Primary resistant n = 144 (41.5%)	DC (CR, PR, SD) n = 203 (58.5%)	
Age distribution					0.383
Median		68 (54.0–74.0)			
< 60	197 (37.2%)	108 (31.1%)	39 (27.1%)	69 (34%)	
60–75	180 (34%)	127 (36.6%)	55 (38.2%)	72 (35.5%)	
> 75	153 (28.8%)	112 (32.3%)	50 (34.7%)	62 (30.5%)	
Gender					0.079
Male	301 (56.8%)	207 (59.7%)	78 (54.2%)	129 (63.5%)	
Female	229 (43.2%)	140 (40.3%)	66 (45.8%)	74 (36.5%)	
Tumour localization♦					0.007
Head and neck	85 (20.6%)	59 (21.5%)	16 (15.1%)	43 (25.4%)	
Trunk	144 (34.9%)	81 (29.5%)	24 (22.6%)	57 (33.7%)	
Extremity	166 (40.2%)	118 (42.9%)	57 (53.8%)	61 (36.1%)	
Other	18 (4.3%)	17 (6.1%)	9 (8.5%)	8 (4.8%)	
Histological subtype♦					0.013
SSM	134 (35.6%)	80 (31.1%)	33 (34%)	47 (29.4%)	
NM	118 (31.4%)	80 (31.1%)	22 (22.7%)	58 (36.3%)	
LMM	17 (4.5%)	14 (5.4%)	1 (1%)	13 (8.1%)	
ALM	37 (9.8%)	32 (12.5%)	16 (16.5%)	16 (10%)	
Mucosal	18 (4.8%)	17 (6.6%)	9 (9.3%)	8 (5.0%)	
Other	52 (13.9%)	34 (13.3%)	16 (16.5%)	18 (11.2%)	
Stage at initial diagnosis♦					0.130
I	89 19.7%)	52 (17.4%)	21 (17.5%)	31 (17.4%)	
II	129 (28.5%)	91 (30.5%)	29 (24.3%)	62 (34.8%)	
III	159 (35.2%)	105 (35.3%)	44 (36.7%)	61 (34.3%)	
IV	75 (16.6%)	50 (16.8%)	26 (21.7%)	24 (13.5%)	
Number of organs with metastases					0.03
1–3	462 (87.2%)	309 (89%)	122 (84.7%)	187 (92.1%)	
> 3	68 (12.8%)	38 (11%)	22 (15.3%)	16 (7.9%)	
Brain metastases					0.901
No brain metastases	404 (76.2%)	283 (81.6%)	117 (81.2%)	166 (81.8)	
Brain metastases	126 (23.8%)	64 (18.4%)	27 (18.8%)	37 (18.2%)	
Liver metastases					0.065
No liver metastases	338 (63.8%)	222 (64%)	84 (58.3%)	138 (68%)	
Liver metastases	192 (36.2%)	125 (36%)	60 (41.7%)	65 (32%)	
BRAF mutation♦					0.529
BRAF mutation	216 (59.7%)	96 (44.9%)	35 (42.2%)	61 (46.6%)	
BRAF wild type	146 (40.3%)	118 (55.1%)	48 (57.8%)	70 (53.4%)	
LDH level♦					0.016
Normal	279 (62.1%)	200 (66.2%)	73 (58.4%)	127 (71.8%)	
Elevated	170 (37.9%)	102 (33.8%)	52 (41.6%)	50 (28.2%)	
S100 level♦					0.000
Normal	230 (51.5%)	168 (54.7%)	51 (40.8%)	117 (64.3%)	
Elevated	217 (48.5%)	139 (45.3%)	74 (59.2%)	65 (35.7%)	

* 8 patients excluded due to lack of information on best response

♣ $\chi$^2^ test performed between primary resistant group and DC group

♦ Patients for which the information was unknown were excluded

The prognostic factors in patients with primary resistance were baseline level of S100 (*p *= 0.003), baseline level of LDH (*p *= 0.007), number of organs with metastases (*p *= 0.024) and presence of liver metastases (*p *= 0.012). Patients with primary resistance had a significantly worse prognosis compared to those that achieved DC: median PFS was 4 months (95% CI 3.62–4.3) for patients with primary resistance and not reached in patients DC.

The median OS was 11 months (95% CI 8.83–13.17) in patients with primary resistance and was not reached in patients with disease control. The 1-y, 2-y and 3-y OS was 43.1% 17% and 10.8% in patients with primary resistance and 91.8%, 80.6% and 64.2% in the group of patients that achieved DC (95% CI 34.5–51.7; 9.9–24.1; 4.3–17.3 and 87.7–95.9; 73.4–87.6; 53.4–75.0, respectively).

There was no difference in terms of survival when the type of first-line immunotherapy (PD-1 monotherapy or CTLA-4 + PD-1) was analyzed: median OS was 26 months for both sub-groups (95% CI 19.7–32.2 and 20.5–31.5, respectively). The 1-y, 2-y and 3-y OS was 71.8%, 53.2%, 41.2% for patients receiving PD-1 monotherapy and 72.8%, 56.2%, 41% for those receiving CTLA-4 + PD-1 (95% CI 64.7–78.9; 45.0–61.4; 32.0–50.4 and 65.0–80.6; 44.8–67.6; 22.6–59.4, respectively).

**Conclusions:** Patients with primary resistance to immunotherapy have a worse prognosis compared to those that achieve disease control. Further research is necessary to earlier identifying these patients and offering other therapeutic options.

##### 17 Modulating the extracellular TCR–CD3 interaction to identify novel immunotherapy targets against melanoma

###### Aswin Natarajan^1^, Yogambigai Velmurugu^1^, Yuan Zhou^2^, Chenghao Ge^2^, Vidushan Nadarajah^1^, Klara Felsovalyi^3^, Timothy J. Cardozo^3^, Clay Bracken^4^, Cheng Zhu^2^, Michelle Krogsgaard^1*^

####### ^1^Department of Pathology and Perlmutter Cancer Center, NYU School of Medicine, New York, NY; ^2^Coulter Department of Biomedical Engineering, Georgia Institute of Technology, Atlanta, GA; ^3^Department of Biochemistry and Molecular Pharmacology, NYU School of Medicine, New York, NY; ^4^Department of Biochemistry, Weill Cornell Medical College, New York, NY

**Correspondence:** Michelle Krogsgaard - Michelle.Krogsgaard@nyulangone.org

*Journal of Translational Medicine* 2020, **18(Supp 1)**:17

**Background:** T cell recognition of antigen and resulting proximal signaling are key steps in the initiation of the adaptive immune response. Identification of the specific extracellular contacts between the T cell receptor (TCR) and CD3 subunits upon recognition of peptide-major histocompatibility complexes (pMHC) gives more precise guidance for immunotherapeutic strategies that modulate T-cell immunity by targeting signaling through the TCR-CD3 complex. Previous studies that targeted the antigen binding site for enhancing T-cell responses to tumor antigens often lead to off-target effects and toxicity.

**Materials and methods:** Recently, we used nuclear magnetic resonance (NMR) spectroscopy, mutational analysis and computational docking to derive a 3D structure of the extracellular TCR-CD3 assembly [1]. Further, biomolecular force probe (BFP) measurements allowed us to determine how 2D affinity and force-modulated TCR-pMHC kinetics depend on TCR-CD3 interaction sites and affect transduction of extracellular pMHC-TCR ligation into T cell function.

**Results:** Based on our TCR-CD3 structural model, we mutated specific TCR-residues (Fig. 1A) that resulted in decreased TCR-CD3 binding (as evident from CD3γε tetramer binding—Fig. 1B) as well as lower cytokine responses (Fig. 1C). However, one Cβ helix 4-F strand mutant, NP202203AA showed higher T cell response (Fig. 1B). This mutant also showed enhanced TCR-pMHC bond lifetime in BFP assays leading to prolonged T cell signaling. Collectively, this data places us in a unique position to translate our findings towards improved immunotherapy strategies.

**Fig. 1:**
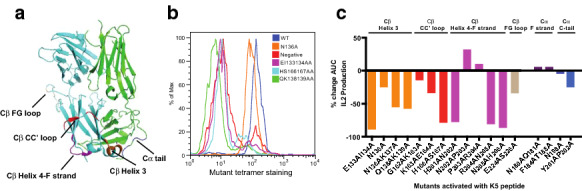
TCR mutations represented in the structure (A); CD3γε tetramer binding to hybridoma T cells (B) and mutant T cell hybridoma activation assay

**Conclusion:** Our hypothesis is that by modulating TCR–CD3 interactions in specific ways, immune-mediated cytotoxicity can be increased without losing specificity for the cancer antigen. To test our hypothesis, we sought to mutate specific TCR-residues that interact with CD3 to increase the affinity of the TCR–CD3 interaction, resulting in better CD3 tetramer binding as well as higher cytokine responses. Previously, we have used structure-based modeling to redesign the antigen binding region of DMF5 TCR (a TCR specific for Mart-1 melanoma antigen) to increase T cell signaling potency [2]. A TCR library for DMF5 TCR was created using site-specific mutagenesis in the Cβ helix 3 and helix 4-F strand regions of the TCR (Fig. 1A) by in vitro combinatorial retroviral TCR display to optimize the TCR–CD3 interaction and to select for mutants with enhanced T-cell effector function. In the future, DMF5 TCR with reengineered CD3 binding regions will be used in tumor rejection in pre-clinical mouse melanoma models for efficacy and toxicity to develop more effective T cell therapies for human targets.

**References**

1. Natarajan A, Nadarajah V, Felsovalyi K, Wang W, Jeyachandran VR, Wasson RA, Cardozo T, Bracken C, Krogsgaard M. Structural model of the extracellular assembly of the TCR–CD3 complex. Cell Rep. 2016; 14: 2833–45.
2. Malecek K, Grigoryan A, Zhong S, Gu WJ, Johnson LA, Rosenberg SA, Cardozo T, Krogsgaard M. Specific increase in potency via structure-based design of a TCR. J Immunol. 2014; 193: 2587–99.

##### 18 Preliminary analysis of the immune modulatory effects of domatinostat, a class I HDAC inhibitor, on tumor immune phenotype in melanoma patients refractory/non-responding to checkpoint inhibitor therapy

###### Anne Catherine Bretz^1^, Tobias Dreker^1^, René Bartz^1^, Frank Hermann^1^, and Svetlana Hamm^1*^

####### ^1^4SC AG, Martinsried, Germany

**Correspondence:** Svetlana Hamm - svetlana.hamm@4SC.com

*Journal of Translational Medicine* 2020, **18(Supp 1)**:18

**Background:** A significant proportion of melanoma patients without as well as with pre-existing immunity fail to respond to checkpoint inhibitor therapy, indicating a therapeutic potential for combining PD-(L)1 therapy with immunomodulating agents in both immunophenotypes. Domatinostat is a class I specific HDAC inhibitor in clinical development in advanced stage melanoma and gastro-intestinal cancer. In PD-(L)1 refractory mouse models without pre-existing immunity domatinostat increased inflammation and expression of genes predictive for PD-1 blockade response. In highly inflamed but exhausted tumors domatinostat beneficially affected the function of cytotoxic T cells within the tumor microenvironment. The combination of domatinostat with PD-(L)1 blockade substantially increased the anti-tumoral effects above the single agent therapies in tumors of both immunophenotypes, displaying greater benefit in highly inflamed but exhausted tumors.

Here, we analyzed the baseline immunophenotypes and the impact of domatinostat mono-therapy on immune scores in melanoma patients refractory/non-responding to prior checkpoint therapy to clinically confirm our preclinical findings for domatinostat.

**Methods:** Progressive, advanced stage melanoma patients with best response (BOR) of PD or SD to prior checkpoint inhibitor therapy were treated with domatinostat as monotherapy in a priming cycle followed by a combination treatment of domatinostat and pembrolizumab (2 mg/kg q3w) until progression or intolerability (SENSITIZE, NCT03278665). Domatinostat was evaluated at three dose levels (100 mg once daily (OD), 200 mg OD, and 200 mg twice daily (bid) in a 14 days on + 7 days off schedule). Tumor biopsies for RNA-Seq analysis were taken at baseline and after the priming cycle (C1D15) to investigate the immune-modulating effect of domatinostat.

**Results:** Analysis of immunophenotype in baseline biopsies of SENSITIZE patient revealed a very high interpatient variability comprising patients with non-inflamed to highly inflamed tumors. The majority of patients had tumors belonging to the highly inflamed, but exhausted immunophenotype. Domatinostat treatment showed a trend to increase the immune-related scores especially in patients with non- or low-inflamed tumors. In patients with high inflammation scores the effect of domatinostat was less consistent. Yet, this patient subpopulation showed the best clinical benefit confirming our pre-clinical observations. Based on murine data we postulate that domatinostat could increase the functionality of exhausted intratumoral T cells.

**Conclusions:** In summary, these data could show that most patients refractory/non-responding to prior checkpoint therapy included into the SENSITIZE trial had an inflamed but exhausted tumor immunophenotype. For domatinostat we could confirm our preclinical hypothesis for a trend to increase the immune scores in non- or low-inflamed tumors and a greater benefit in the highly inflamed tumor patients.

**Acknowledgements:** We thank all the patients and investigators (P. Ascierto, J.C. Hassel, D. Schadendorf, C. Berking, R. Gutzmer, T. Eigentler, B. Schilling) participating in the SENSITIZE clinical trial.

## Melanoma Bridge

### Emergent strategies session

#### Oral communications

##### 19 Pharmacodynamic effect of tebentafusp (TCR–CD3 bispecific) on peripheral cytokines and association with overall survival in patients with advanced melanoma

###### Mark R Middleton^1^, Neil Mathew Steven^2^, Thomas Jeff Evans^3^, Jeffry R Infante^4^, Omid Hamid^5^, Alexander Noor Shoushtari^6^, Pippa Corrie^7^, Alan Anthoney^8^, Avinash Gupta^9^, Victoria K Woodcock^1^, Cheryl McAlpine^10^, Mario Sznol^11^

####### ^1^Department of Oncology, Medical Sciences Division, University of Oxford, Oxford, UK; ^2^Institute of Immunology and Immunotherapy, College of Medical and Dental Sciences, University of Birmingham, Birmingham, UK; ^3^Institute of Cancer Sciences, University of Glasgow, Glasgow, UK; ^4^Janssen, Philadelphia, PA, USA; ^5^ImmunoOncology, The Angeles Clinic and Research Institute, Los Angeles, CA, USA; ^6^Memorial Sloan Kettering Cancer Center, New York, NY, USA; ^7^Cambridge University Hospitals, Cambridge, UK; ^8^Leeds Teaching Hospitals NHS Trust, Leeds, UK; ^9^The Christie NHS Foundation Trust, Manchester, UK; ^10^Immunocore Ltd, Oxford, UK; ^11^Yale Cancer Center, Yale School of Medicine, Yale, CT, USA

**Correspondence:** Mark R Middleton - mark.middleton@oncology.ox.ac.uk

*Journal of Translational Medicine* 2020, **18(Supp 1)**:19

**Background:** ImmTAC molecules are unique TCR-anti-CD3 bispecifics that redirect T cells against intracellular antigens. Tebentafusp (IMCgp100), an ImmTAC targeting melanocyte-expressed gp100 antigen, has demonstrated monotherapy activity in advanced melanoma and can cause rash and cytokine-mediated AEs, hypothesized to be on-target (gp100) or effector (CD3) mediated. A preclinical MoA for T cell bispecifics suggests chemokine CXCL10 redirection of CXCR3+ T cells from blood into antigen-positive tissues; this has not been clinically validated.

**Methods:** 84 HLA-A2+ pts with advanced melanoma (n = 61 cutaneous [CM], n = 19 uveal [UM], n = 4 other) received tebentafusp. Serum (n = 40) and PBMC (n = 22) samples were taken pre- and post-infusion to analyze changes in cytokines and circulating T cells. Pre- (n = 16) and post-treatment (n = 11) tumor biopsies were analyzed by IHC for CD3, PD-L1 and gp100 expression; tumor RNA (n = 12) was analyzed for gene expression.

**Results:** Tebentafusp induced a transient increase in IFNγ-inducible cytokines, most prominently CXCL10. A greater increase in serum CXCL10 was associated with longer OS (p = 0.0002), tumor shrinkage (p = 0.003), and greater transient reduction in peripheral CXCR3+ CD8+ T cells (p = 0.001). Reduction in CXCR3+ CD8+ T cells also trended with longer OS (p = 0.02), and tumor shrinkage (p = 0.03).

3/16 pre-treatment biopsies had < 1% gp100 expression (all progressive disease). 8/11 biopsies post-tebentafusp had increased CD3+ T cells compared with matched pre-treatment samples (associated with baseline gp100 but not PD-L1 expression). Based on tumor biopsy gene expression analysis, tebentafusp increased T cell markers, IFNγ-inducible and cytotoxicity-related genes.

**Conclusions:** The association of clinical benefit with increased serum CXCL10 and decreased peripheral CXCR3+ T cells supports the MoA of tebentafusp-induced T cell redirection and activation. Tumor biopsy results support tebentafusp redirection of T cells to antigen-positive tumor. A Phase II trial in CM (NCT02535078), a Phase I/II trial in UM (NCT02570308), and a Pivotal RCT in UM (NCT03070392) are ongoing.

**Trial Registration:** NCT01211262

##### 20 Updated data from IMPemBra, a phase 2 Study Comparing Pembrolizumab (PEM) with Intermittent/short‐term dual MAPK pathway inhibition (MAPKi, dabrafenib + trametinib, D + T) plus PEM in patients harboring the BRAFV600 mutation

###### EA Rozeman^1^, K. Sikorska^2^, L Grijpink-Ongering^2^, B Heeres^3^, B van de Wiel^4^, A Sari^2^, H Mallo^1^, S Adriaansz^1^, W Uyterlinde^1^, J Lijnsvelt^1^, L Pronk^2^, JW de Groot^5^, S Wilgenhof^1^, M Vollebergh^1^, JV van Thienen^1^, JBAG Haanen^1^, and CU Blank^1^

####### ^1^Medical Oncology Department, Netherlands Cancer Institute/Antoni van Leeuwenhoek hospital (NKI-AVL), Amsterdam, Netherlands; ^2^Department of Biometrics, Netherlands Cancer Institute/Antoni van Leeuwenhoek hospital (NKI-AVL), Amsterdam, Netherlands; ^3^Department of Radiology, Netherlands Cancer Institute/Antoni van Leeuwenhoek hospital (NKI-AVL), Amsterdam, Netherlands; ^4^Department of Pathology, Netherlands Cancer Institute/Antoni van Leeuwenhoek hospital (NKI-AVL), Amsterdam, Netherlands; ^5^Department of Medical Oncology, ISALA, Zwolle, the Netherlands

**Correspondence:** Elisa A. Rozeman - l.rozeman@nki.nl

*Journal of Translational Medicine* 2020, **18(Supp 1)**:20

**Background:** Continuous combination of MAPKi and anti-PD-(L)1 is currently tested in several trials to improve outcome of BRAFV600 mutated melanoma patients (pts). However, a major obstacle for continuous combination is the high frequency of grade 3/4 treatment-related adverse events (TRAE). In a preclinical model we showed that short‐time MAPKi induces T cell infiltration and is synergistic with anti-PD-1. In pts we found increased T cell infiltration upon D + T after short-term MAPKi, while this was frequently below baseline levels after > 2 weeks (W) MAPKi. The aim of this phase 2b study was to identify the optimal duration of D + T in combination with PEM.

**Methods:** Treatment-naïve BRAFV600E/K mutant advanced melanoma pts (n = 32) started PEM 200 mg Q3W and were randomized in W6 to continue PEM only (cohort 1), or to receive in addition intermittent D 150 mg BID + T 2 mg QD for 2× 1 W (cohort 2), 2× 2 W (cohort 3), or continuous for 6 W (cohort 4). All cohorts continued PEM for up to 2 years. Primary endpoints were safety and treatment-adherence. Secondary endpoints were objective response rate (ORR, RECIST 1.1) at week 6, 12, 18 compared to baseline and PFS.

**Results:** The data from the first 26 pts completed the first 18 W were presented at ESMO 2018. Grade 3/4 TRAE within the first 18 W were observed 0%, 14%, 33%, and 50% of pts in cohort 1, 2, 3, and 4, respectively. All planned D + T was given in 86%, 50%, and 33% of pts in cohort 2, 3, and 4. ORR at W6, W12, and W18 were 29%, 57%, and 57% in cohort 1, 29%, 71%, and 71% in cohort 2, 33%, 50%, and 83% in cohort 3 and 0%, 50%, and 50% in cohort 4.

We will present the updated ORR and toxicity data from all 32 pts. In addition, we will present for the first time PFS and OS data from the complete four cohorts with a median FU of 18 months.

**Conclusion:** The ESMO 2018 IMPemBra data indicated that PEM + intermittent D + T for 2× 1 W or 2× 2 W are promising combinations in terms of safety and feasibility, warranted to be tested in subsequent trials.

**Clinical trial information:** NCT02977052

##### 21 Clinical activity of BEMPEG plus NIVO in previously untreated patients with metastatic melanoma: updated results from the phase 1/2 PIVOT-02 study

###### Adi Diab^1^, Igor Puzanov^2^, Michele Maio^3^, Brendan Curti^4^, Mehmet Bilen^5^, Karl Lewis^6^, Scott Tykodi^7^, Gregory Daniels^8^, Alexander Spira^9^, Chantale Bernatchez^1^, Salah Eddine Bentebibel^1^, Michael Wong^1^, James Larkin^10^, Ewa Kalinka-Warzocha^10^, Sunny Xie^12^, Sue Currie^12^, Ute Hoch^12^, Wei Lin^12^, Mary Tagliaferri^12^, Stina Singel^12^, Michael Hurwitz^13^

####### ^1^MD Anderson Cancer Center, Houston, Texas, USA; ^2^Roswell Park Cancer Institute, Buffalo, New York, USA; ^3^Azienda Ospedaliera Universitaria Senese, Siena, Italy; ^4^Providence Portland Medical Center, Portland, Oregon, USA; ^5^Emory University Hospital, Atlanta, Georgia, USA; ^6^University of Colorado, Denver, Colorado, USA; ^7^Seattle Cancer Care Alliance, Seattle, Washington, USA; ^8^University of California, San Diego, California, USA; ^9^Virginia Cancer Specialists, Fairfax, Virginia, USA; ^10^The Royal Marsden, London, UK; ^11^Instytut Medyczny Santa Familia, Lodz, Poland; ^12^Nektar Therapeutics, San Francisco, California, USA; ^13^Yale School of Medicine, New Haven, Connecticut, USA

**Correspondence:** Igor Puzanov - Igor.Puzanov@roswellpark.org

*Journal of Translational Medicine* 2020, **18(Supp 1)**:21

**Background:** Although checkpoint inhibitor (CPI) therapy has emerged as an effective treatment option for various cancers, there is an unmet need for therapies to produce more durable and deeper responses in metastatic melanoma. Safety and clinical activity of bempegaldesleukin (BEMPEG; NKTR-214), a CD-122 preferential IL-2 pathway agonist, plus the anti-PD1 CPI nivolumab (NIVO), was evaluated in PIVOT-02 (NCT02983045), a multicenter phase 1/2 study in multiple solid tumor settings. At SITC 2018, PIVOT-02 reported encouraging preliminary clinical activity and safety data in metastatic melanoma (ORR, 53%; CR, 24%) [1, 2]. We plan to report updated results in 1L metastatic melanoma patients, and the first report of PFS.

**Methods:** 41 patients with previously untreated stage IV metastatic melanoma received ≥ 1 dose of BEMPEG (0.006 mg/kg) + NIVO (360 mg) q3w. Patients were categorized by PD-L1 status. Response was assessed every 3 cycles by RECISTv1.1. Per protocol, ORR was evaluated in the efficacy-evaluable population (≥ 1 post-baseline scan) by independent central radiology review (N = 38; 3 patients, non- efficacy-evaluable: 1 unrelated treatment-emergent AE; 2 patient decisions). Baseline immunohistochemistry (IHC) analysis for PD-L1 was performed (using Dako PD-L1 IHC 28-8 pharmDx) and defined as PD-L1 negative (< 1% tumor cell expression) and PD-L1 positive (≥ 1% tumor cell expression). Safety and tolerability were assessed by CTCAEv4.03.

**Results:** At a median follow-up of 12.7 months*, 38 patients were evaluable for efficacy. Table 1 shows BEMPEG plus NIVO was associated with clinical activity regardless of PD-L1 status. Confirmed ORR was 53% (20/38), and 34% (13/38) achieved a complete response. 42% (16/38) had 100% reduction in target lesions. Median time to response was 2 months, and median time to complete response was 7 months. Median duration of response was not reached (range: 11mo-NR). BEMPEG plus NIVO was well tolerated, with TRAEs similar to those previously reported, with 14.6% (6) patients experiencing a ≥ Grade 3 TRAE, and 9.8% (4) discontinuing treatment due to any TRAE. As of July 10, 2019, all 10 patients reported on treatment on March 29th, 2019 remain on treatment or achieved maximum response. At time of presentation, updated clinical results, including PFS, with ~ 18 months of follow-up will be reported.

**Table 1:** Clinical activity and deepening of response of efficacy-evaluable population at 12.7 month median follow-up^2^ (N = 38)*

Clinical response	Number of patients (%)
Confirmed ORR (CR + PR)	20 (53%)
Complete response (CR)	13 (34%)
DCR (CR + PR + SD**)	28 (74%)
ORR in PD-L1 negative (n = 14)	6 (43%)
ORR, PD-L1 positive (n = 21)	13 (62%)
ORR, PD-L1 unknown (n = 3)	1 (33%)
ORR, LDH > ULN (n = 11)	5 (45%)
ORR, liver metastases (n = 10)	5 (50%)

*Data as of March 29, 2019 cut-off date. **Disease control rate, defined as complete response or partial response or stable disease for at least 8 weeks

**Conclusions:** BEMPEG plus NIVO is associated with robust clinical activity in 1 L metastatic melanoma, as demonstrated by a high rate of durable responses that deepened over time. Based on these data, the FDA granted Breakthrough Therapy Designation for this combination therapy for patients with untreated unresectable or metastatic melanoma, and a Phase 3 trial evaluating the combination of BEMPEG plus NIVO vs NIVO alone in this setting is currently enrolling (NCT03635983).

**References**

1. Diab A, Tykodi S, Curti B, et al. Immune monitoring after NKTR‐214 plus nivolumab (PIVOT‐02) in previously untreated patients with metastatic stage IV melanoma oral presentation at SITC; November 7–11, 2018; Washington, DC, USA. Abstract #O4
2. Hurwitz M, Cho DC, Balar, AV, et al. Baseline tumor-immune signatures associated with response to bempegaldesleukin (NKTR-214) and nivolumab. J Clin Oncol. 2019; 37:(suppl;2623).

##### 22 Phase Ib/II study combining the HDAC inhibitor domatinostat with anti-PD-1 pembrolizumab in patients with advanced melanoma refractory/non-responding to prior checkpoint inhibitor therapy

###### Paolo A. Ascierto^1^, Jessica C. Hassel^2^, Carola Berking^3^, Thomas Eigentler^4^, Ralf Gutzmer^5^, Bastian Schilling^6^, Frank Hermann^7^, René Bartz^7^ and Dirk Schadendorf^8^

####### ^1^Istituto Nazionale Tumori IRCCS Fondazione “G. Pascale”, Melanoma Cancer Immunotherapy and Development Therapeutics Unit; Naples, Italy; ^2^University Hospital Heidelberg, Department of Dermatology and National Center for Tumor Diseases; Heidelberg, Germany; ^3^University Hospital Munich (LMU), Department of Dermatology; Munich, Germany; ^4^University Hospital Tuebingen, Center for Dermatooncology, Department of Dermatology; Tuebingen, Germany; ^5^Medizinische Hochschule Hannover, Department of Dermatology and Allergy, Skin Cancer Center Hannover; Hannover, Germany; ^6^University Hospital Würzburg, Department of Dermatology, Venereology and Allergology; Würzburg, Germany; ^7^4SC AG, Planegg-Martinsried, Germany; ^8^University Hospital Essen, Department of Dermatology; Essen, Germany

**Correspondence:** Paolo A. Ascierto - paolo.ascierto@gmail.com

*Journal of Translational Medicine* 2020, **18(Supp 1)**:22

**Background:** Anti-PD-1 alone or in combination with anti-CTLA4 is the current standard of care for advanced unresectable or metastatic cutaneous melanoma. Despite remarkable response rates, a significant proportion of patients does not achieve any or only timely limited disease control, are refractory or do not respond to anti-PD-1-containing therapy. Epigenetic modulation, and Histone Deacetylase (HDAC) inhibition in particular is intended to enhance the immunogenicity of the tumor, alter the tumor microenvironment and thus increase the chance of clinical activity to such immunotherapy, having the potential of a new clinical approach to overcome tumor escape mechanisms.

**Methods:** In an open label Phase Ib/II multi-center study (‘SENSITIZE’) we investigate the combination of the selective class I HDAC inhibitor domatinostat and pembrolizumab in progressive patients with unresectable or metastatic cutaneous melanoma with best response of progressive disease (PD) or stable disease (SD) to prior checkpoint inhibitor therapy. These advanced stage melanoma patients were treated with domatinostat (orally) with increasing dose levels in the first 3 different dose cohorts in combination with pembrolizumab in a modified “rolling six” study design to evaluate the safety and tolerability of the combination treatment. The trial is designed to determine the optimal biological dose (dose escalation) and dosing schedule (dose optimization). Tumor assessments are performed every 12 weeks and evaluated per irRECIST. Sequential tumor biopsies are taken for immunohistochemical and gene expression analysis and peripheral blood for pharmacokinetic (PK) analysis.

On 15-July-2019, 23 patients with progressive, unresectable or metastatic cutaneous melanoma with best response of PD or SD to prior anti-PD-1 therapy have been treated in the SENSITIZE study.

**Results:** Domatinostat in combination with pembrolizumab is safe and well tolerated up to 200 mg twice daily (BID) in a 14 + 7 dosing schedule). 4/23 patients experienced grade 3 treatment related AEs and no grade 4 TRAEs occurred, furthermore no increase in frequency or intensity of immune-related AEs were observed. Additionally, first signs of clinical activity have been observed showing a trend towards dose-dependency of domatinostat with a disease control in 4 out of 7 patients (highest dose cohort, 200 mg BID).

**Conclusion:** Preliminary analyses of tumor biopsies suggest a trend towards a domatinostat-induced change in immunological tumor patterns corroborating findings from earlier preclinical work. These observations in this advanced, heavily pre-treated patient population warrant further development of domatinostat in combination with anti-PD-(L)1 antibodies to sensitize the tumor, the tumor microenvironment and the patient’s immune response for synergistic anti-tumor activity (Fig. 1).

**Fig. 1:**
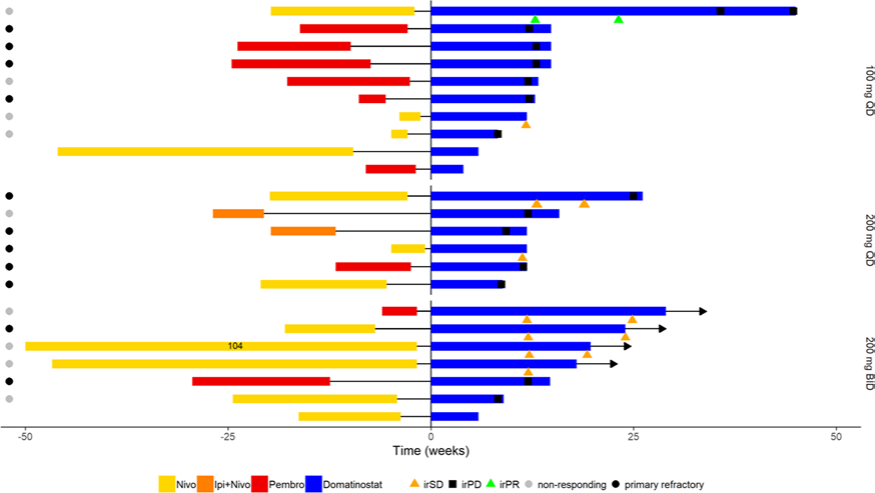
Last prior therapy and preliminary efficacy SENSITIZE in 3 different dose cohorts

**Trial Registration:** ClinicalTrials.gov: NCT03278665

##### 23 Safety and Efficacy of TRIplet combination of Nivolumab (N) with Dabrafenib (D) and Trametinib (T) [TRIDeNT] in Patients (pts) with BRAF-mutated Metastatic Melanoma (MM): A Single Center Phase II Study

###### Elizabeth M. Burton^1^, Rodabe N. Amaria, Isabella Claudia Glitza, Michael Shephard, Adi Diab, Denai Milton, Sapna Pradyuman Patel, Jennifer McQuade, Michael K. K. Wong, Patrick Hwu, Jennifer Wargo, Michael A. Davies, Hussein Tawbi

####### ^1^Director of research planning & development for melanoma clinical, translational, and prevention research efforts, Director of research planning & development for melanoma clinical, translational, and prevention research efforts, Houston, TX, USA

**Correspondence:** Elizabeth M. Burton - emburton@mdanderson.org

*Journal of Translational Medicine* 2020, **18(Supp 1)**:23

**Background:** Although targeted therapies (TT) and immunotherapies (IMT) have improved survival for pts with BRAF V600 mutated stage IV MM, many pts progress and will ultimately die from this disease. Preclinical data has shown that BRAF inhibition (BRAFi) in BRAF-mutated tumors is associated with increased T cell infiltration, supporting the rationale for a clinical combinatorial approach with IMT. Although there are multicentered trials ongoing evaluating this combinatorial approach for pts with untreated MM, there are no approved therapies for pts after TT and IMT failure. Notably, pts with untreated brain metastases (BM) are often excluded from such trials. We hypothesized that N in combination with DT is safe and will demonstrate clinical activity in BRAF-mutated pts refractory to PD1 therapy and in pts with BM.

**Methods:** We report a single arm phase II study (NCT02910700) of NDT in pts with BRAF-mutated, unresectable stage III or stage IV MM. Prior IMT is allowed, but pts who have received BRAF/MEKi are ineligible. Pts with untreated BM and asymptomatic or mildly symptomatic/requiring stable or decreasing steroids (up to PO dexamethasone of 8 mg or equivalent) are also allowed. Pts received 3 mg/kg Q2wks of N (later amended to 480 mg q4wks), 150 mg BID of D and 2 mg QD of T, all starting on Day 1. The primary objective of this study is to determine safety and efficacy (ORR by RECIST 1.1) of the NDT combination. This study was continuously monitored for safety and futility. Tissue and blood-based samples to assess for correlative studies are also collected.

**Results:** Following a 6 pts safety run-in with no observed DLTs, 26 pts received NDT—16 pts were PD1 refractory, 10 were PD-1 naive. 9 of these 26 pts had BM. Of the 22 pts evaluable for response, 17 achieved PR and 3 CR (ORR 91%). 12 PD1 refractory were evaluable for response; 2 achieved CR and 9 PR (ORR 83%). 67% of the evaluable pts with BM achieved an intracranial response, including 2 CRs. Although the median PFS for all pts was ~ 8 months, the median OS was not reached. 65% of pts experienced treatment related grade 3/4 AEs, but only 3 pts discontinued due to toxicities.

**Conclusions:** NDT is well-tolerated and shows promising clinical activity in pts with IMT refractory disease and with BM. There were no significant differences in outcomes between pts with and without BM. Further translational investigation to better delineate mechanisms of response are ongoing.

## Melanoma Bridge 2019

### Poster

#### 24 Relationship between clinical efficacy and AEs of tebentafusp, a novel bispecific TCR-anti-CD3, in patients with advanced melanoma

##### Mark R Middleton^1^, Neil Mathew Steven^2^, Thomas Jeff Evans^3^, Jeffry R Infante^4^, Mario Sznol^5^, Omid Hamid^6^, Alexander Noor Shoushtari^7^, Alan Anthoney^8^, Avinash Gupta^9^, Victoria K Woodcock^1^, Shaad Abdulla^10^, Pippa Corrie^11^

###### ^1^Department of Oncology, Medical Sciences Division, University of Oxford, Oxford, UK; ^2^Institute of Immunology and Immunotherapy, College of Medical and Dental Sciences, University of Birmingham, Birmingham, UK; ^3^Institute of Cancer Sciences, University of Glasgow, Glasgow, UK; ^4^Janssen, Philadelphia, PA, USA; ^5^Yale Cancer Center, Yale School of Medicine, Yale, CT, USA; ^6^ImmunoOncology, The Angeles Clinic and Research Institute, Los Angeles, CA, USA; ^7^Memorial Sloan Kettering Cancer Center, New York, NY, USA; ^8^Leeds Teaching Hospitals NHS Trust, Leeds, UK; ^9^The Christie NHS Foundation Trust, Manchester, UK; ^10^Immunocore Ltd, Oxford, UK; ^11^Cambridge University Hospitals, Cambridge, UK

####### **Correspondence:** Mark R Middleton - mark.middleton@oncology.ox.ac.uk

*Journal of Translational Medicine* 2020, **18(Supp 1)**:24

**Background:** Bispecific antibodies have shown activity in hematologic (heme) but not solid tumors. ImmTAC molecules are unique TCR-anti-CD3 bispecifics that redirect T cells against intracellular antigens. Tebentafusp (IMCgp100), an ImmTAC targeted against melanocyte-associated lineage antigen gp100, has shown monotherapy responses in advanced melanoma with associated immune changes. Tebentafusp causes rash and cytokine-mediated AEs, hypothesized to be on-target (gp100) or effector (CD3) mediated. We explored clinical and biological characteristics of pts associated with treatment benefit.

**Materials and methods:** 84 HLA-A2 positive advanced melanoma pts received tebentafusp on study IMCgp100-01 in 13 dose escalation cohorts. Efficacy was assessed by Kaplan–Meier survival and treatment related AEs (TRAE) reported by CTCAE v4.0. Serum samples evaluated changes in cytokines. A multivariate analysis investigated the relationship between efficacy and safety variables.

**Results:** Demographics: 73% cutaneous (CM), 23% uveal (UM) primaries; 51% LDH > ULN; 25% received prior anti-PD(L)1.

83 (99%) pts had ≥ 1 TRAE; most commonly in skin (rash 82%, pruritus 69%) or cytokine-mediated (pyrexia 57%); the majority were Grade 1–2 and occurred and resolved within first 3 doses. The 2 most frequent Grade ≥ 3 TRAEs were rash (26%) and lymphopenia (13%). Tebentafusp induced transient increases in peripheral cytokines (peaking Day 1–2) that attenuated with subsequent doses; cytokine-mediated AE had similar kinetics.

1-yr OS was 65% (95% CI 48–78). In multivariate analysis, longer OS was associated with: LDH ≤ ULN (p = 0.002) and any-grade rash occurring within 21 days (p = 0.003); melanoma primary site and prior anti-PD-(L)1 did not significantly affect outcome. In exploratory analyses, longer OS associated with lower baseline serum IL-6 (n = 43) or TNFα (n = 44).

**Conclusions:** Tebentafusp is a first-in-class, TCR-based bispecific with monotherapy efficacy in advanced melanoma. AEs were manageable and consistent with MoA. Association between tebentafusp efficacy and on-target TRAEs, previously reported for bispecifics to heme lineage antigens, is now recognized for solid tumor lineage antigens. Pivotal studies in UM are ongoing.

**Trial Registration:** NCT01211262

#### 25 MicroRNA-193a family as potential clinical biomarker and therapeutic agent in advanced Melanoma

##### Sara Carpi^1^, Beatrice Polini^1^, Leena Ylösmäki^2^, Erkko Ylösmäki^2^, Vincenzo Cerullo^2^, Antonella Romanini^3^, Paola Nieri^1^

###### ^1^Laboratory of Molecular Pharmacology, Department of Pharmacy, University of Pisa, Italy; ^2^Laboratory of Immunovirotherapy, Drug Research Program, Faculty of Pharmacy, University of Helsinki, Viikinkaari 5E, 00790, Helsinki, Finland; ^3^University Hospital of Pisa, Oncology Section, Pisa, Italy

####### **Correspondence:** Sara Carpi - sara.carpi@unipi.it

*Journal of Translational Medicine* 2020, **18(Supp 1)**:25

**Background:** The relevant role played by microRNAs (miRNAs) in cancer, as in other diseases, make them possible new drugs or drug targets as well as diagnostic and prognostic disease biomarkers. MiR-193a acts as potential tumour suppressor in malignant pleural mesothelioma, gastric and non-small cell lung cancer and it regulates drug and chemoradiation resistance in bladder and oesophageal cancer, respectively [1]. As regards melanoma, actually a study evaluating the expression of miR-193a in cutaneous melanoma tissues and cell lines [2] and a pilot investigation from our laboratory on its levels in plasma of melanoma patients compared to healthy controls have been realized [3].

Nevertheless, no data are reported on the role of miR-193a on the control of melanoma cell proliferation and metastasis. Here, effect of miR-193a ectopic expression was investigated in vitro and in vivo melanoma model. Parallely, its expression in plasma exosomes derived from stage IV melanoma patients was analysed in order to confirm its role as diagnostic biomarker.

**Materials and methods:** In order to evaluate the tumour suppressor role of miR-193a in melanoma cells, we studied its influence on intracellular pathways regulating survival, proliferation, apoptosis and migration, such as MAPK/ERK, and PI3K/Akt, and on markers involved in epithelial-mesenchymal transition (EMT). The in vivo miR-193a anti-cancer effects were evaluated in the murine B16.OVA melanoma model by using a viral (Modified Vaccinia Ankara, MVA) platform. Exosomes were isolated from plasma samples of melanoma patients and healthy donors, and their miR-193a levels were determined via quantitative real-time PCR.

**Results:** In vitro experiments showed a significant decrease of melanoma cell viability and migration and an increase of apoptosis in transfected cells. Furthermore, a significant decrease in B-Raf protein levels and in phosphorylation of Akt and Erk proteins was observed, suggesting the miR-193a ability to interfere with cell proliferation and survival. Vimentin and E-Cadherin transcriptional and protein levels were significantly modulated, indicating the potential of this miRNA to contrast EMT. A significant decrease of the miR-193a target PD-L1 in the in vivo murine melanoma model, suggests an efficient delivery of the functional miR by the viral platform. Finally, a statistically significant decrease in the miR-193a levels was observed in exosome-derived plasma of metastatic melanoma patients compared to healthy donors.

**Conclusions:** Our data suggest that miR193a represents a potential therapeutic agent reducing melanoma progression and confirm its diagnostic biomarker role in this cancer type. Experiments aimed at deepened its anti-melanoma potential in the in vivo model are ongoing.

**References**

1. Yu T, Li J, Yan M, et al. MicroRNA-193a-3p and -5p suppress the metastasis of human nonsmall- cell lung cancer by downregulating the ERBB4/PIK3R3/mTOR/S6K2 signaling pathway. Oncogene. 2015;34(4):413–23.
2. Caramuta S, Egyházi S, Rodolfo M, et al. MicroRNA expression profiles associated with mutational status and survival in malignant melanoma. J Invest Dermatol. 2010;130(8):2062–70.
3. Fogli S, Polini B, Carpi S, et al. Identification of plasma microRNAs as new potential biomarkers with high diagnostic power in human cutaneous melanoma. Tumour Biol. 2017;39(5):1010428317701646.

#### 26 Correlation between CT scan findings at 3 and 6 months and pattern of response, progression-free survival and overall survival in advanced metastatic melanoma patients treated by anti-PD1 monotherapy: a single institution retrospective study

##### Pietro Quaglino, Alessandra Testi, Paolo Fava, Chiara Astrua, Lucia Stigliano, Matteo Brizio, Elena Marra, Simone Ribero, Marco Rubatto, Luca Tonella, Maria Teresa Fierro

###### Dermatologic Clinic, Department of Medical Sciences, University of Turin Medical School

####### **Correspondence:** Pietro Quaglino - pietro.quaglino@unito.it

*Journal of Translational Medicine* 2020, **18(Supp 1)**:26

**Introduction:** Treatment with anti-PD1 induces responses in about 40% of advanced metastatic melanoma with median response induction time of 2 to 3 months. However, late responses are described, as well as atypical responses. In the real life, the decision whether to prolong or when to stop treatment in patients with slow progressing disease is still a challenge.

**Objectives:** To evaluate anti-PD1 clinical activity in this real-life setting; to summarise the findings of CT-scans performed at 3 and 6 months after treatment starting; to correlate 3 and 6 month CT findings with BOR, Progression-Free Survival (PFS) and Overall Survival (OS).

**Materials and methods:** Retrospective single centre study which included 112 consecutive advanced metastatic melanoma treated as first line with anti-PD1 as monotherapy since 2015. Clinical features, stage of disease, number of metastatic sites, LDH values were evaluated at baseline before treatment. CT-scan findings were evaluated at 3 and 6 months and categorised as follows: reduction/regression of lesions; stable lesions; increase of dimensions of pre-existing lesions; occurrence of new lesions; both increase of pre-existing and occurrence of new lesions.

**Results:** The BOR was CR in 15.2% and PR in 20.5% of patients. The response rate was 35.7%, the clinical benefit was confirmed in 49.7% of patients. CT findings at 3 months were significantly correlated with BOR: 35/43 patients (81%) with reduction or stable lesions achieved a clinical benefit whilst only 8/43 (19%) developed a PD; on the other hand, among patients with new lesions, increase of pre-existing or both, only 6/53 (11%) developed a clinical benefit whilst 47 (89%) progressed (p = 0.0001). The same figures were obtained when analysing CT scans at 6 months. Atypical responses occurred in 6 out of 112 patients (5.3%). These patients were characterised by < 3 metastatic sites (6/6), good PS (6/6), normal LDH values (5/6), no brain metastases and prevalence of M1a/b score (4/6). Median OS for the entire patient cohort was 1.7 years (4 months–4 years) with a 3-year OS of 35%, median PFS was 10.5 months. Median OS calculated since the 3-month CT-scan showed significant benefit in patients with stable/regressing lesions with respect to the others (median: 1.9 years vs 10.3 months; p = 0.001).

**Conclusion:** The results of this study suggest the relevant predictive value of 3-month CT-scan findings which are correlated with disease outcome in terms of BOR, clinical benefit and OS. In patients with dimension increase or new lesions at 3 or 6 months CT-scan, treatment continuation should be considered in cases with favourable PS and low tumour burden.

This work was funded with the TESEO project Medicina di precisione nelle neoplasie mediante omica e big data, Progetto Strategico di Eccellenza Dipartimentale, DSM, UNITO.

